# Machine learning-based integration develops an immune-derived lncRNA signature for improving outcomes in colorectal cancer

**DOI:** 10.1038/s41467-022-28421-6

**Published:** 2022-02-10

**Authors:** Zaoqu Liu, Long Liu, Siyuan Weng, Chunguang Guo, Qin Dang, Hui Xu, Libo Wang, Taoyuan Lu, Yuyuan Zhang, Zhenqiang Sun, Xinwei Han

**Affiliations:** 1 Department of Interventional Radiology, The First Affiliated Hospital of Zhengzhou University, Zhengzhou, Henan China; 2 Interventional Institute of Zhengzhou University, Zhengzhou, Henan China; 3 Interventional Treatment and Clinical Research Center of Henan Province, Zhengzhou, Henan China; 4 Department of Hepatobiliary and Pancreatic Surgery, The First Affiliated Hospital of Zhengzhou University, Zhengzhou, Henan China; 5 Department of Endovascular Surgery, The First Affiliated Hospital of Zhengzhou University, Zhengzhou, Henan China; 6 Department of Colorectal Surgery, The First Affiliated Hospital of Zhengzhou University, Zhengzhou, Henan China; 7 Department of Cerebrovascular Disease, Zhengzhou University People’s Hospital, Zhengzhou, Henan China

**Keywords:** Cancer, Computational biology and bioinformatics, Drug discovery, Immunology, Molecular biology

## Abstract

Long noncoding RNAs (lncRNAs) are recently implicated in modifying immunology in colorectal cancer (CRC). Nevertheless, the clinical significance of immune-related lncRNAs remains largely unexplored. In this study, we develope a machine learning-based integrative procedure for constructing a consensus immune-related lncRNA signature (IRLS). IRLS is an independent risk factor for overall survival and displays stable and powerful performance, but only demonstrates limited predictive value for relapse-free survival. Additionally, IRLS possesses distinctly superior accuracy than traditional clinical variables, molecular features, and 109 published signatures. Besides, the high-risk group is sensitive to fluorouracil-based adjuvant chemotherapy, while the low-risk group benefits more from bevacizumab. Notably, the low-risk group displays abundant lymphocyte infiltration, high expression of CD8A and PD-L1, and a response to pembrolizumab. Taken together, IRLS could serve as a robust and promising tool to improve clinical outcomes for individual CRC patients.

## Abstract (web summary)

Identification of long non-coding RNA (lncRNA) signatures could be used to improve cancer clinical outcome. Here the authors developed a machine learning-based integrative procedure to construct a consensus immune-related lncRNA signature to predict prognosis, recurrence and treatment benefits in colorectal cancer.

## Introduction

Colorectal cancer (CRC) is characterised by strong heterogeneity and aggressiveness, with high prevalence and mortality^1^. This mortality can be largely attributed to disease progression and inadequate treatment^2^. Hence, early intervention for “high-risk” CRC is crucial to improve clinical outcomes. In the clinical setting, the American Joint Committee on Cancer (AJCC) classification is a conventional tool to evaluate the risk and treatment demand of a specific patient based on clinical stage. However, the limitations of the current staging system may hamper its ability to provide optimal clinical care to patients, as clinical decisions to conduct adjuvant chemotherapy (ACT) are primarily determined by clinicopathological staging, without regard to molecular biological characteristics^3^. This insufficient approach might give rise to latent overtreatment or undertreatment. Recently, immune checkpoint inhibitors (ICIs) have emerged as a revolutionary modality of cancer immunotherapy that functions by targeting immune checkpoints^4^. However, to date, only a subset of patients has yielded considerable benefit from ICI treatment. The candidate biomarkers that facilitate the clinical selection of patients for ICI treatment include programmed death-ligand 1 (PD-L1) expression, tumour mutation burden (TMB), neoantigen load (NAL), and mismatch repair deficiency (dMMR)/microsatellite instability-high (MSI-H), but these approaches are limited by spatiotemporal heterogeneity, moderate accuracy, or small percentage populations^5–7^. Thus, in the era of individualised treatment, identifying reliable biomarkers for optimising the prognosis and benefits of drug therapies in CRC is imperative.

CRC is a complex disease with both inter- and intratumour heterogeneity. An ideal biomarker should have homogenous expression within and between tumour tissues to perform robustly across all patients. Therefore, a multigene panel might be a promising method to address this heterogeneity^2^. With the advancements in bioinformatics technology, a multitude of prognostic gene signatures have been developed^2,8–11^. Signatures integrated by multigene profiles, particularly messenger RNAs (mRNAs) or microRNAs (miRNAs), were discovered and validated as candidate biomarkers in CRC^9,10,12^. Nevertheless, due to underutilized data information, inappropriate machine learning methods, lack of rigorous verification by different cohorts, and no clinical testing, multigene expression signatures are usually difficult to apply in clinical settings^13–15^. Newly discovered noncoding RNAs, called long noncoding RNAs (lncRNAs), are defined as >200 nucleotides in length and have mRNA-like transcripts with no protein-coding capacity^16^. Thus, it is necessary to incorporate lncRNAs into preclinical models to develop prognostic biomarkers. Indeed, accumulating studies have revealed that lncRNAs are closely implicated in tumourigenesis, progression, prognosis, and drug resistance and sensitivity^17^. Of note, emerging evidence has also reported that lncRNAs play fundamental roles in inflammatory responses; the development, differentiation, and effector function of immune cells; the tumour immune microenvironment; and cancer immunotherapy^18–20^.

In this work, we attempted to apply immune-related lncRNAs to develop and validate a risk stratification signature in 2509 CRC patients from 17 independent public datasets and a clinical in-house cohort to assess the prognosis, recurrence, and benefits of fluorouracil-based ACT, bevacizumab, and ICI treatment in CRC. This work may help optimise precision treatment and further improve the clinical outcomes of CRC patients.

## Results

### Development and validation of immune infiltration consensus clusters

**Fig. 1:**
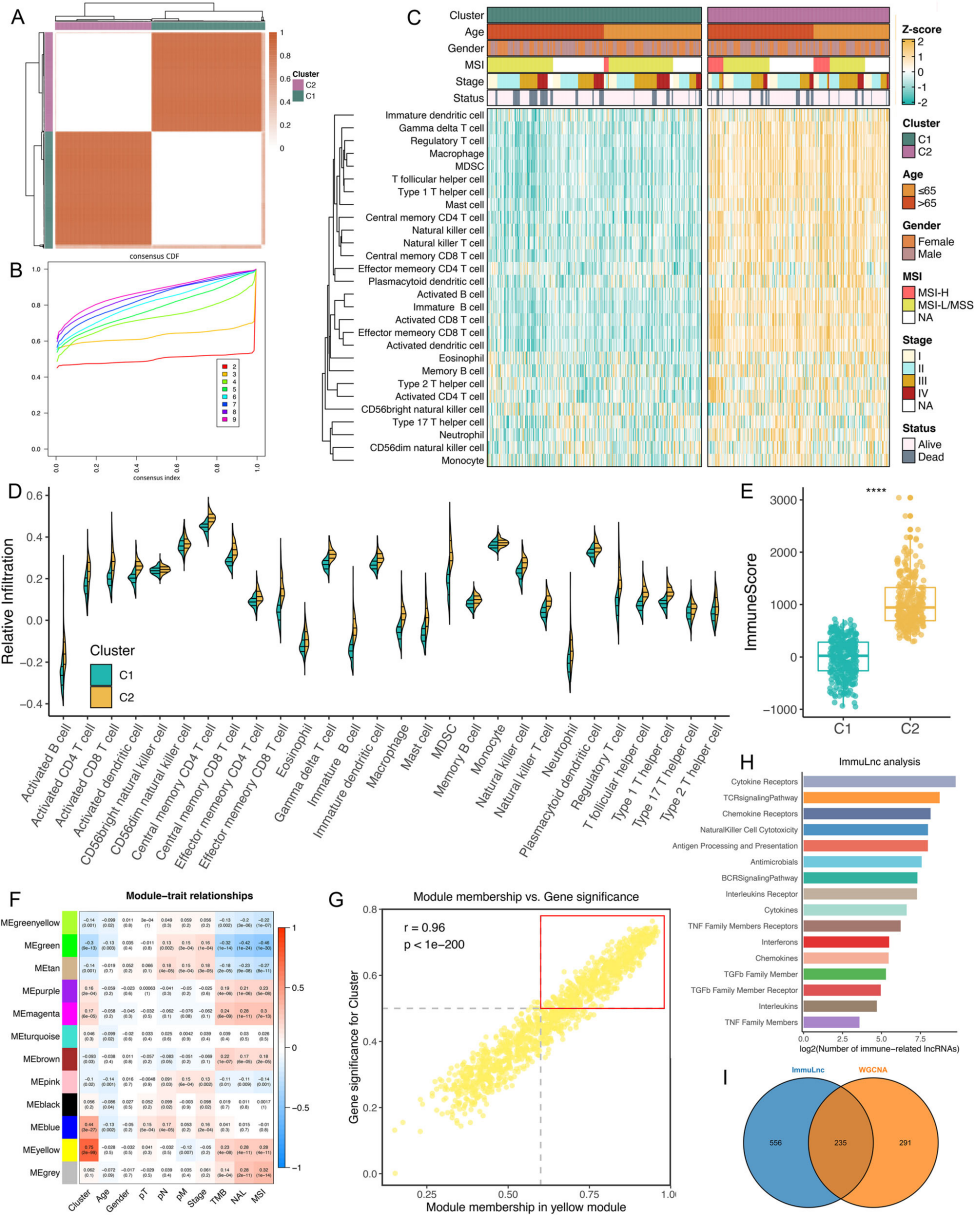
Identification of immune-related lncRNAs via two algorithms. **A** The consensus score matrix of all samples when k = 2. A higher consensus score between two samples indicates they are more likely to be grouped into the same cluster in different iterations. **B** The CDF curves of consensus matrix for each k (indicated by colours). **C** The infiltration abundance of 28 immune cell subsets evaluated by ssGSEA for two clusters. **D** The distribution of 28 immune cell subsets infiltration between two clusters. **E** The distribution of immune score inferred by ESTIMATE algorithm between two clusters in the TCGA-CRC cohort (*n* = 584, *P* = 5.22e−113). Statistic test: two-sided unpaired *t* test. In boxplot graphs centre line indicates median, bounds of box indicate 25th and 75th percentiles, and whiskers indicate minimum and maximum. ****P < 0.0001. **F** Correlation analysis between module eigengenes and clinical traits. **G** The high correlation between GS and MM in the yellow module (*P* = 0). Dots within the red rectangle were defined as immune-related lncRNAs, with both high GS and MM. Statistic test: Pearson’s correlation coefficient, two-sided unpaired *t* test. **H** ImmLnc identified a total of 791 lncRNAs significantly associated with immune‐related pathways. **I** The overleaping lncRNAs between WGCNA and ImmLnc.

The overall design of this study is displayed in Supplementary Fig. 1. According to 28 immune cells infiltration assessed by single-sample gene set enrichment analysis (ssGSEA)^21^, we performed a consensus cluster analysis^22^, in which all CRC samples were initially divided into *k* (*k* = 2–9) clusters. The cumulative distribution function (CDF) curves of the consensus score matrix and proportion of ambiguous clustering (PAC) statistic^23^ indicated that the optimal number was obtained when *k* = 2 (Fig. 1A, B and Supplementary Fig. 2A). The same result was achieved from Nbclust testing (Supplementary Fig. 2B). The two consensus clusters (C1 and C2) demonstrated significant differences in immune infiltration, with C2 having a markedly higher overall infiltration abundance than C1 (Fig. 1C, D). Thus, we defined C1 as “immune-cold” tumours and C2 as “immune-hot” tumours. To ensure that the two consensus clusters were not biased by the analytical algorithm, six other algorithms, including TIMER, quanTIseq, MCP-counter, xCell, EPIC, and ESTIMATE, were used to verify the stability and robustness of the ssGSEA results (Supplementary Fig. 2C and Fig. 1E).

### Identification of lncRNA modules derived from immune infiltration patterns

In the weighted correlation network analysis (WGCNA) procedure, the soft threshold β was set to 9 (no scale *R*^*2*^ = 0.910), which provided a suitable power value for coexpression network construction (Supplementary Fig. 2D). Then, 12 modules were identified, as indicated by different colours. The eigengene (first principal component of gene expression within a module) was considered as the representative of the module. The heatmap revealed the eigengene adjacency of modules (Supplementary Fig. 2E). Furthermore, the correlations between modules and clinical traits, such as immune clusters, age, gender, T stage, N stage, M stage, AJCC stage, TMB, NAL, and microsatellite state, were calculated. The highest correlation in the module-trait relationship was observed between the yellow module and immune clusters (Fig. 1F). In the yellow module, the correlation coefficient between gene significance (GS) and module membership (MM) reached 0.96, which suggested that the quality of lncRNA module construction was superior (Fig. 1G). To identify hub lncRNAs derived from immune infiltration patterns within the yellow module, 526 lncRNAs with GS > 0.5 and MM > 0.6 were considered hub immune-related lncRNAs (Fig. 1G).

### Immune-related lncRNAs generated from the ImmLnc pipeline

ImmLnc systematically deduces candidate lncRNA regulators of immune‐related pathway activity from lncRNA and gene expression profiles^9,18^. One assumption is that, if a specific lncRNA plays critical roles in immune regulation, then its related genes should be enriched in the top or bottom of immune‐related pathways. By virtue of the ImmLnc pipeline, we identified 791 immune-related lncRNAs (Supplementary Data 1). A high number of lncRNAs were correlated with the “cytokine receptors”, “TCR signalling pathway”, “chemokine receptors”, “natural killer cell cytotoxicity”, and “antigen processing and presentation” pathways (Fig. 1H). With the intersection of WGCNA results, a total of 235 overlapping lncRNAs were extracted for subsequent analysis (Fig. 1I).

### Integrative construction of a consensus signature

**Fig. 2:**
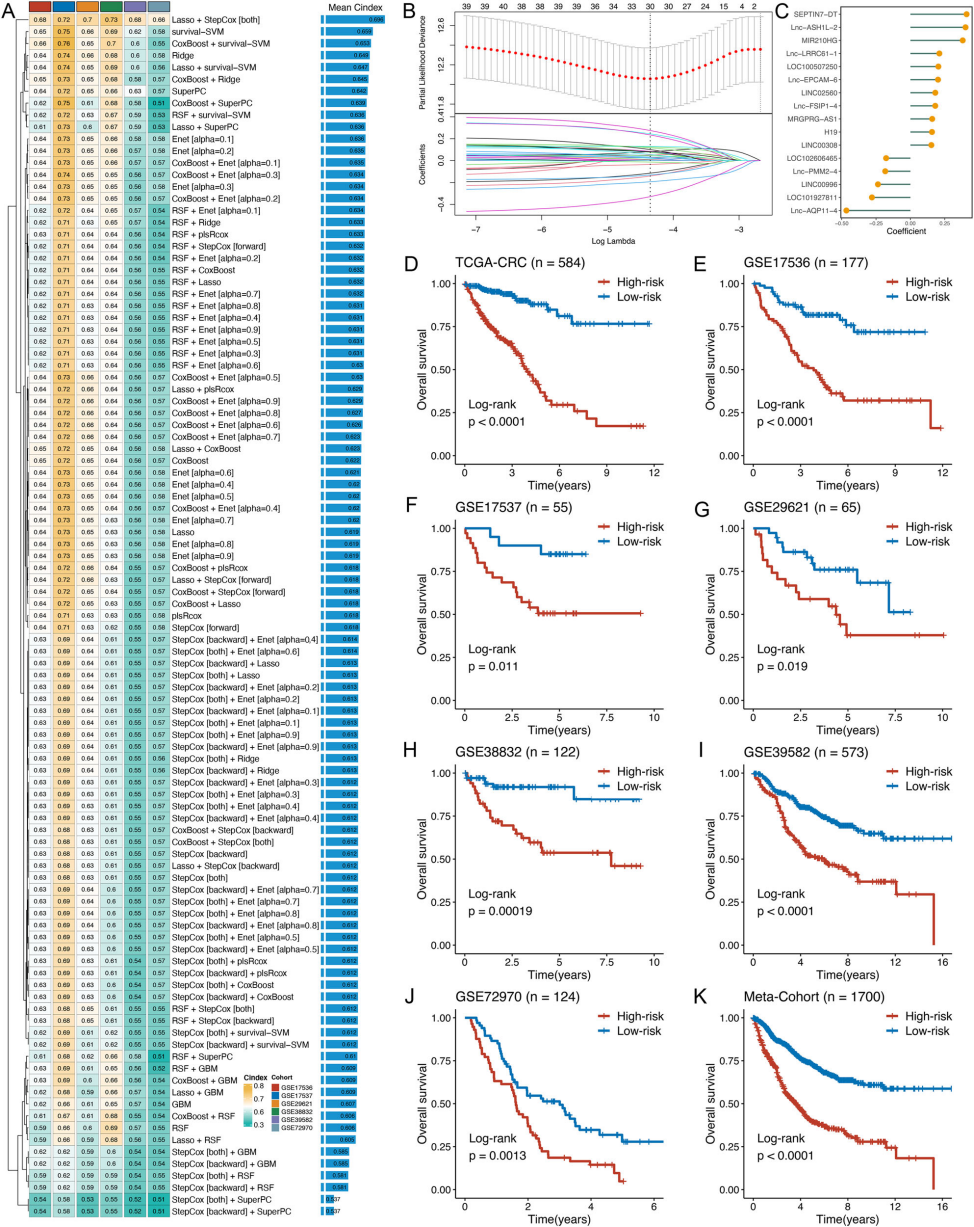
A consensus IRLS was developed and validated via the machine learning-based integrative procedure. **A** A total of 101 kinds of prediction models via LOOCV framework and further calculated the C-index of each model across all validation datasets. **B** In the TCGA-CRC cohort (*n* = 584), the determination of the optimal λ was obtained when the partial likelihood deviance reached the minimum value, and further generated Lasso coefficients of the most useful prognostic genes. Data are presented as mean ± 95% confidence interval [CI]. **C** Coefficients of 16 lncRNAs finally obtained in stepwise Cox regression. **D**–**K** Kaplan–Meier curves of OS according to the IRLS in TCGA-CRC (log-rank test: *P* = 9.16e−19) (**D**), GSE17536 (log-rank test: *P* = 2.79e−7) (**E**), GSE17537 (log-rank test: *P* = 0.011) (**F**), GSE29621 (log-rank test: *P* = 0.019) (**G**), GSE38832 (log-rank test: *P* = 1.87e−4) (**H**), GSE39582 (log-rank test: *P* = 2.06e−10) (**I**), GSE72970 (log-rank test: *P* = 0.0013) (**J**), and meta-cohort (log-rank test: *P* = 5.18e−35) (**K**).

Based on the expression profiles of 235 immune-related lncRNAs, univariate Cox analysis identified 43 prognostic lncRNAs (Supplementary Fig. 2F). These 43 lncRNAs were subjected to our machine learning-based integrative procedure to develop a consensus immune-related lncRNA signature (IRLS). In the TCGA-CRC dataset, we fitted 101 kinds of prediction models via the LOOCV framework and further calculated the C-index of each model across all validation datasets (Fig. 2A and Supplementary Data 2). Interestingly, the optimal model was a combination of Lasso and stepwise Cox (direction = both) with the highest average C-index (0.696), and this combination model had a leading C-index in all validation datasets (Fig. 2A). In the Lasso regression, the optimal λ was obtained when the partial likelihood deviance reached the minimum value based on the LOOCV framework (Fig. 2B). Thirty lncRNAs with nonzero Lasso coefficients were subjected to stepwise Cox proportional hazards regression, which identified a final set of 16 lncRNAs (Fig. 2C).

Next, a risk score for each patient was calculated using the expression of 16 lncRNAs weighted by their regression coefficients in a Cox model (Fig. 2C). All patients were assigned into high- and low-risk groups according to the optimal cut-off value determined by the *survminer* package. As illustrated in Fig. 2D–J, patients in the high-risk group had significantly dismal overall survival (OS) relative to the low-risk group in the TCGA-CRC training dataset and six validation datasets (all *P* < 0.05). The meta-cohort that combined all samples also showed the same trend (*P* < 0.05) (Fig. 2K). Multivariate Cox regression demonstrated that IRLS remained statistically significant (all *P* < 0.05) after adjusting for available clinical traits, such as age; gender; T, N, M, and AJCC stage; TMB; NAL; microsatellite state; ACT; and TP53, KRAS, or *BRAF* mutations, which suggested that IRLS is an independent risk factor for OS (Supplementary Fig. 3). Subsequently, we further assessed the predictive value of IRLS for RFS in 11 datasets. Kaplan–Meier analysis revealed a consistent trend across all cohorts, with patients in the high-risk group having unfavourable relapse-free survival (RFS) (Supplementary Fig. 4). Notably, two of these cohorts were not statistically significant, possibly due to their small sample size (Supplementary Fig. 4). The meta-cohort displayed a dramatic RFS difference between the two groups (Supplementary Fig. 4). However, multivariate Cox regression indicated that IRLS remained statistically significant for RFS in only 3 of the 11 cohorts (Supplementary Fig. 5). Hence, for RFS, IRLS had a certain degree of predictive value, but it was not an independent prognostic factor.

### Evaluation of the IRLS model

**Fig. 3:**
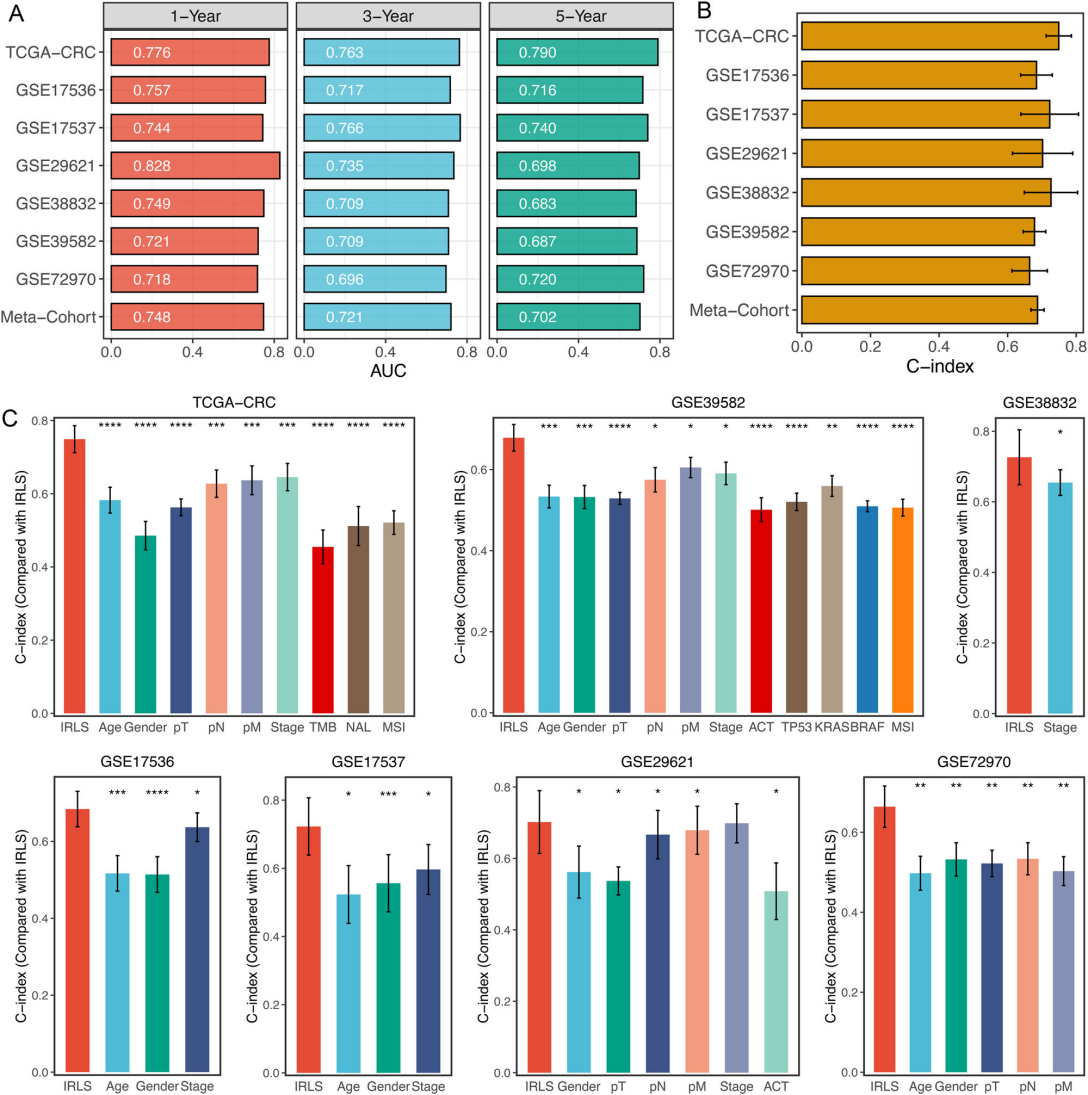
Evaluation of the IRLS model. **A**Time-dependent ROC analysis for predicting OS at 1, 3, and 5 years. **B** C-index of IRLS across all datasets. **C** The performance of IRLS was compared with other clinical and molecular variables in predicting prognosis. Statistic tests: two-sided z-score test. Data in (**B**, **C**) are presented as mean ± 95% confidence interval [CI]. *P < 0.05; **P < 0.01; ***P < 0.001; ****P < 0.0001.

ROC analysis measured the discrimination of IRLS, with 1-, 3-, and 5-year AUCs of 0.776, 0.763, and 0.790 in TCGA-CRC; 0.757, 0.717, and 0.716 in GSE17536; 0.744, 0.766, and 0.740 in GSE17537; 0.828, 0.735, and 0.698 in GSE29621; 0.749, 0.709, and 0.683 in GSE38832; 0.721, 0.709, and 0.687 in GSE39582; 0.718, 0.696, and 0.720 in GSE72970; and 0.748, 0.721, and 0.702 in meta-cohort, respectively (Fig. 3A and Supplementary Data 3). The C-index [95% confidence interval] was 0.749 [0.712–0.786], 0.684 [0.638–0.730], 0.723 [0.639–0.807], 0.702 [0.614–0.790], 0.726 [0.649–0.804], 0.678 [0.646–0.711], 0.664 [0.612–0.716], and 0.687 [0.668–0.706] in the eight cohorts, respectively (Fig. 3B and Supplementary Data 3). Furthermore, we also calculated two other time-independent indicators, integrated AUC (iAUC) and integrated Brier score (IBS) (Supplementary Fig. 6 and Supplementary Data 3). All these indicators suggested that IRLS had stable and robust performance in multiple independent cohorts. A previous study reported that clinical characteristics (e.g. AJCC stage) and molecular alterations (e.g. microsatellite state, KRAS mutations) were also used to assess the prognosis of CRC in clinical practice^24^. Therefore, we compared the performance of IRLS with other clinical and molecular variables in predicting prognosis. As displayed in Fig. 3C, IRLS had distinctly superior accuracy than the other variables including age; gender; T, N, M, and AJCC stage; TMB; NAL; microsatellite state; ACT; and TP53, KRAS, or BRAF mutations (all *P* < 0.05, except for comparison between IRLS and AJCC stage in GSE29621). An interesting idea is to combine IRLS with commonly used clinical traits to further elevate clinical utility. AJCC stage is a commonly used tool for the clinical management of CRC, and multivariate Cox regression analysis of AJCC stage was statistically significant across multiple cohorts. Thus, we further explored the performance of IRLS + Stage. As shown in Supplementary Fig. 7, we found that the performance of IRLS + Stage was significantly better than that of IRLS or AJCC stage alone in multiple datasets. These results led us to conclude that the combination of IRLS and AJCC stage may further improve the predictive ability of our model.

### Comparison of gene expression-based prognostic signatures in CRC

**Fig. 4:**
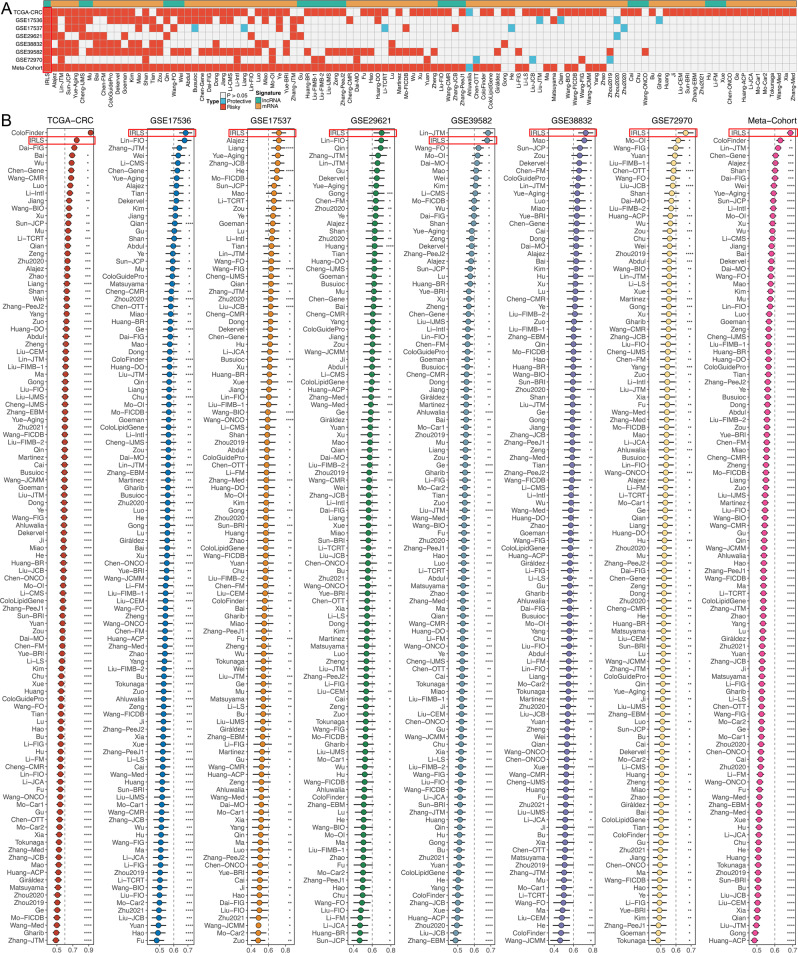
Comparison of gene expression-based prognostic signatures in CRC. **A** Univariate Cox regression analysis of IRLS and 109 published signatures in TCGA-CRC, GSE17536, GSE17537, GSE29621, GSE38832, GSE39582, GSE72970, and meta-cohort. **B** C-index analysis IRLS and 109 published signatures in TCGA-CRC (*n* = 584), GSE17536 (*n* = 177), GSE17537 (*n* = 55), GSE29621 (*n* = 65), GSE38832 (*n* = 122), GSE39582 (*n* = 573), GSE72970 (*n* = 124), and meta-cohort (*n* = 1700). Statistic tests: two-sided z-score test. Data are presented as mean ± 95% confidence interval [CI]. *P < 0.05; **P < 0.01; ***P < 0.001; ****P < 0.0001.

Recently, with developments in next-generation sequencing and big-data technologies, a considerable number of prognostic and predictive gene expression signatures have been developed based on machine learning^25^. To compare the performance of IRLS with other signatures, we comprehensively retrieved published signatures. The miRNA signatures were excluded owing to the severe lack of miRNA information in validation datasets annotated by GPL570. Ultimately, 109 signatures (including mRNA and lncRNA signatures) were enroled (Supplementary Data 4). These signatures were associated with various biological processes, such as immune response, autophagy, ferroptosis, stemness, epithelial–mesenchymal transition, Toll-like receptor signalling, hypoxia, glycolysis, lipogenesis, vitamin D, epigenetics, N6-methyladenosine, ageing, WNT, and drug sensitivity. We performed univariate Cox regression across all datasets for each signature and observed that only our model was significantly associated with prognosis in all cohorts (Fig. 4A), which demonstrated the stability of IRLS. Furthermore, the C-index of IRLS was compared with other signatures; notably, IRLS displayed better performance in every dataset than almost all models (Fig. 4B). We noticed that most models performed well in their own training dataset and a few external datasets (e.g. Chen-Gene, Dai-FIG) but performed weakly in other datasets (Fig. 4B)^26,27^. This may be due to the poor generalisability of the model derived by overfitting. Our signature was reduced dimensionally by two machine learning algorithms and therefore had better extrapolation potential.

### Validation in a clinical in-house cohort

**Fig. 5:**
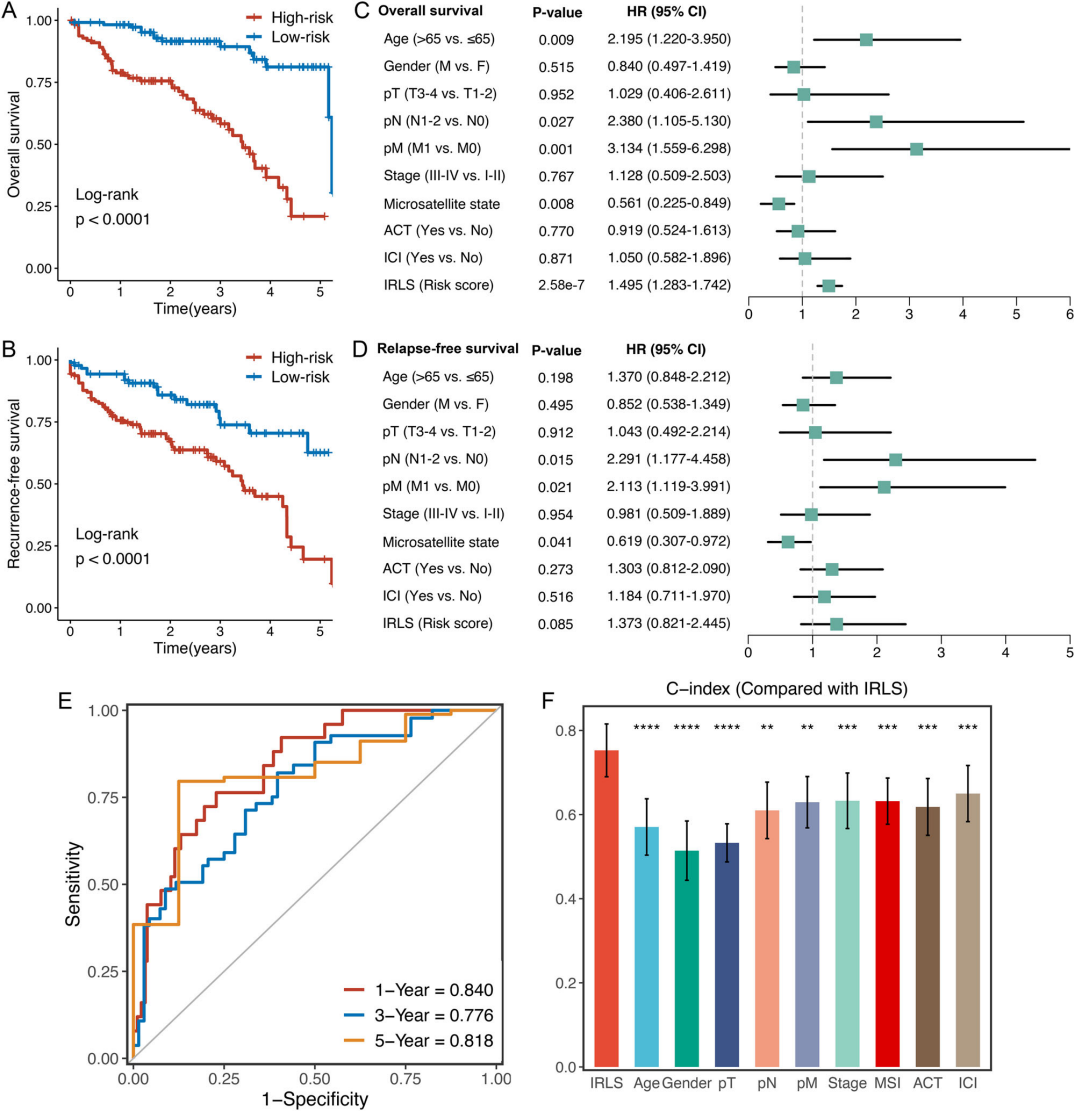
Validation in a clinical in-house cohort. **A**, **B** Kaplan–Meier curves of OS (log-rank test: *P* = 1.93e−9) (**A**) and RFS (log-rank test: *P* = 5.23e−5) (**B**) according to the IRLS. **C**, **D** Multivariable Cox regression analysis of OS (**C**) and RFS (**D**) in our cohort (*n* = 232). Statistic test: two-sided Wald test. Data are presented as hazard ratio (HR) ± 95% confidence interval [CI]. **E** Time-dependent ROC analysis for predicting OS at 1, 3, and 5 years. **F** The performance of IRLS was compared with other clinical and molecular variables in predicting prognosis in our cohort (*n* = 232). Statistic tests: two-sided z-score test. Data are presented as mean ± 95% CI. **P < 0.01; ***P < 0.001; ****P < 0.0001.

To further verify the performance of our IRLS model in a clinically translatable tool, we next evaluated the expression of these lncRNAs in a clinical cohort of 232 CRC patients by conducting qRT-PCR assays. Consistently, Kaplan–Meier analysis demonstrated that patients with high IRLS exhibited dramatically worse OS and RFS (*P* < 0.0001) (Fig. 5A, B). After controlling for confounding variables (including age, gender, T stage, N stage, M stage, AJCC stage, microsatellite state, chemotherapy, and ICI treatment), the IRLS model remained statistically significant for OS instead of RFS (Fig. 5C, D), which was consistent with the above results. ROC analysis showed a superior accuracy of IRLS: the AUCs for predicting OS at 1, 3, and 5 years were 0.840, 0.776, and 0.818, respectively (Fig. 5E). Similarly, the C-index reached 0.765 (95% CI = 0.691–0.839). In addition, we compared the predictive superiority of IRLS with other clinical features and observed that IRLS maintained optimal performance (Fig. 5F). Collectively, the results from a clinical in-house cohort supported our discovery and in silico validation cohort findings, which validated and confirmed that our IRLS model was quite robust and can serve as an independent predictor of prognosis in CRC.

### Predictive value of fluorouracil-based ACT and bevacizumab benefits

**Fig. 6:**
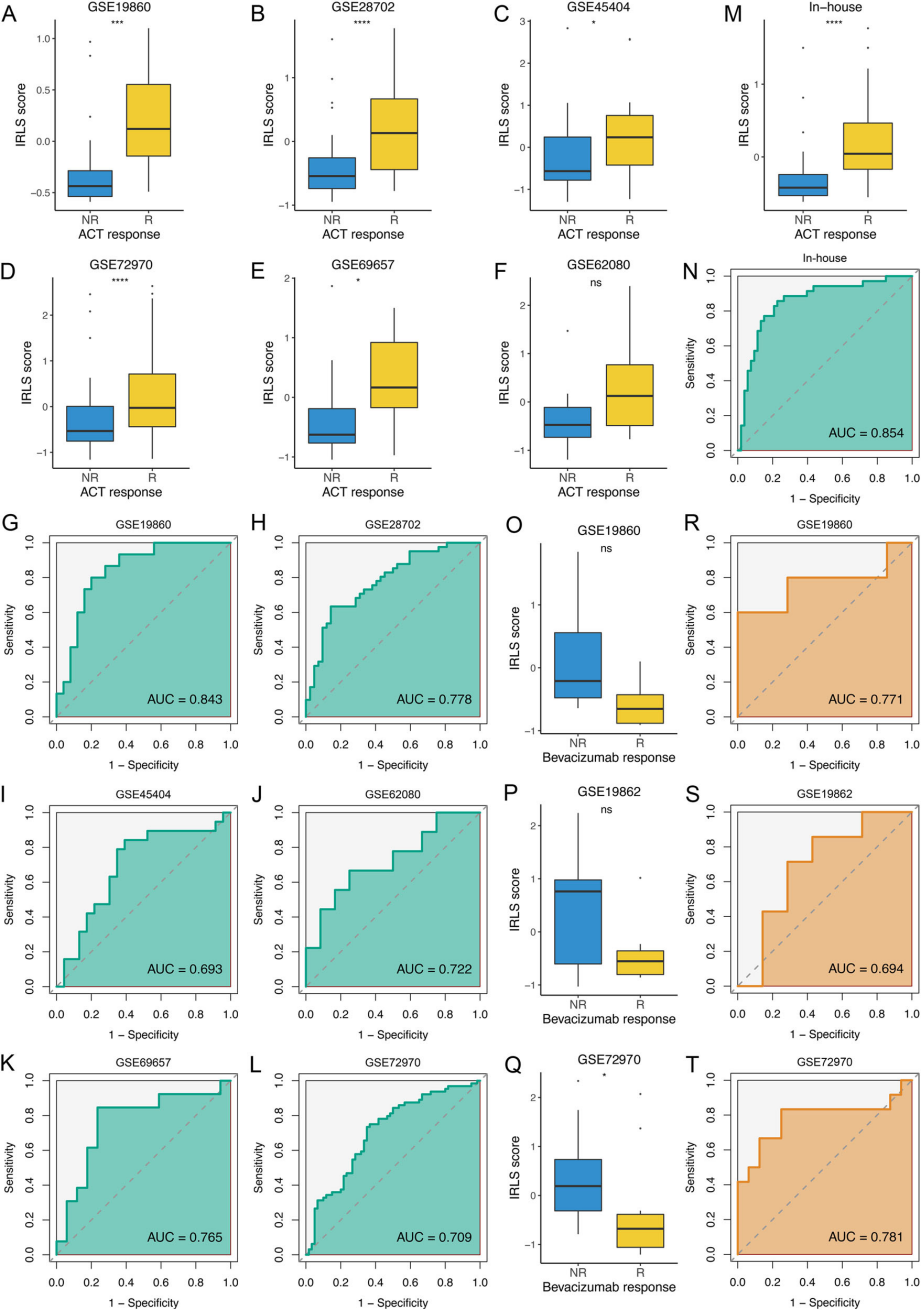
Predictive value of fluorouracil-based ACT and bevacizumab benefits. **A**–**F** The distribution of IRLS score between responders and nonresponders of fluorouracil-based ACT in GSE19860 (*n* = 40, *P* = 1.70e–4) (**A**), GSE28702 (*n* = 83, *P* = 1.42e−5) (**B**), GSE45404 (*n* = 42, *P* = 0.033) (**C**), GSE72970 (*n* = 124, *P* = 5.29e−5) (**D**), GSE69657 (*n* = 30, *P* = 0.015) (**E**), and GSE62080 (*n* = 21, *P* = 0.095) (**F**). Statistic tests: two-sided *t* test. **G**-**L** ROC curves of IRLS to predict the benefits of fluorouracil-based ACT in GSE19860 (**G**), GSE28702 (**H**), GSE45404 (I), GSE62080 (**J**), GSE69657 (**K**), and GSE72970 (**L**). **M** The distribution of IRLS score between responders and nonresponders of fluorouracil-based ACT in in-house cohort (*n* = 88, *P* = 7.64e−6). Statistic test: two-sided *t* test. **N** ROC curves of IRLS to predict the benefits of fluorouracil-based ACT in in-house cohort. **O**–**Q** The distribution of IRLS score between responders and nonresponders of bevacizumab in GSE19860 (*n* = 12, *P* = 0.106) (**O**), GSE19862 (*n* = 14, *P* = 0.318) (**P**), and GSE72970 (*n* = 28, *P* = 0.011) (**Q**). Statistic tests: two-sided *t* test. **R**–**T** ROC curves of IRLS to predict the benefits of bevacizumab in GSE19860 (**R**), GSE19862 (**S**), and GSE72970 (**T**). In boxplot graphs (**A**–**F**, **M**, **O**–**Q**) centre line indicates median, bounds of box indicate 25th and 75th percentiles, and whiskers indicate minimum and maximum. ^ns^*P* > 0.05; *P < 0.05; ***P < 0.001; ****P < 0.0001.

Accumulating evidence has revealed that lncRNAs are implicated in sensitivity and resistance to fluorouracil-based ACT and bevacizumab^18,28–30^. Herein, we further assessed the predictive value of IRLS for quantifying fluorouracil-based ACT and bevacizumab benefit. Six datasets treated with fluorouracil-based ACT were enroled, which included 180 nonresponders and 160 responders. We found that responders presented a significantly higher IRLS score than nonresponders in GSE19860, GSE28702, GSE45404, GSE69657, and GSE72970 (all *P* < 0.05) (Fig. 6A–E). Of note, responders had a trend toward higher IRLS in GSE62080, but this was not significant (*P* = 0.091) (Fig. 6F), which might be due to the small sample size (*n* = 21). ROC analysis demonstrated that IRLS could accurately predict the benefit of fluorouracil-based ACT, with high AUCs in GSE19860 (0.843), GSE28702 (0.778), GSE45404 (0.693), GSE69657 (0.765), GSE72970 (0.709), and GSE62080 (0.722) (Fig. 6G–L). In our in-house cohort, a total of 88 patients received fluorouracil-based ACT, of which 35 patients were included in the responder group (CR, *n* = 11; PR, *n* = 24) and 53 patients in the nonresponder group (SD, *n* = 32; PD, *n* = 21). Likewise, a higher IRLS was displayed in the responder group (Fig. 6M), and IRLS could also markedly discriminate responders from nonresponders of fluorouracil-based ACT in our cohort (AUC = 0.854) (Fig. 6N).

Subsequently, three datasets (GSE19860, GSE19862, and GSE72970), including 30 nonresponders and 24 responders to bevacizumab, were also collected. In contrast to fluorouracil-based ACT alone, patients sensitive to bevacizumab exhibited a lower IRLS level in GSE19860 (*P* = 0.075), GSE19862 (*P* = 0.112), and GSE72970 (*P* = 0.011) (Fig. 6O–Q). The AUCs of IRLS for predicting the benefit of bevacizumab were 0.771, 0.694, and 0.781 in three independent datasets (Fig. 6R–T). This suggested that IRLS also had a robust performance for bevacizumab. Taken together, patients with high IRLS tended to be sensitive to fluorouracil-based ACT and resistant to bevacizumab, while patients with low IRLS tended to be sensitive to bevacizumab and resistant to fluorouracil-based ACT.

### Implications of IRLS for ICI treatment

**Fig. 7:**
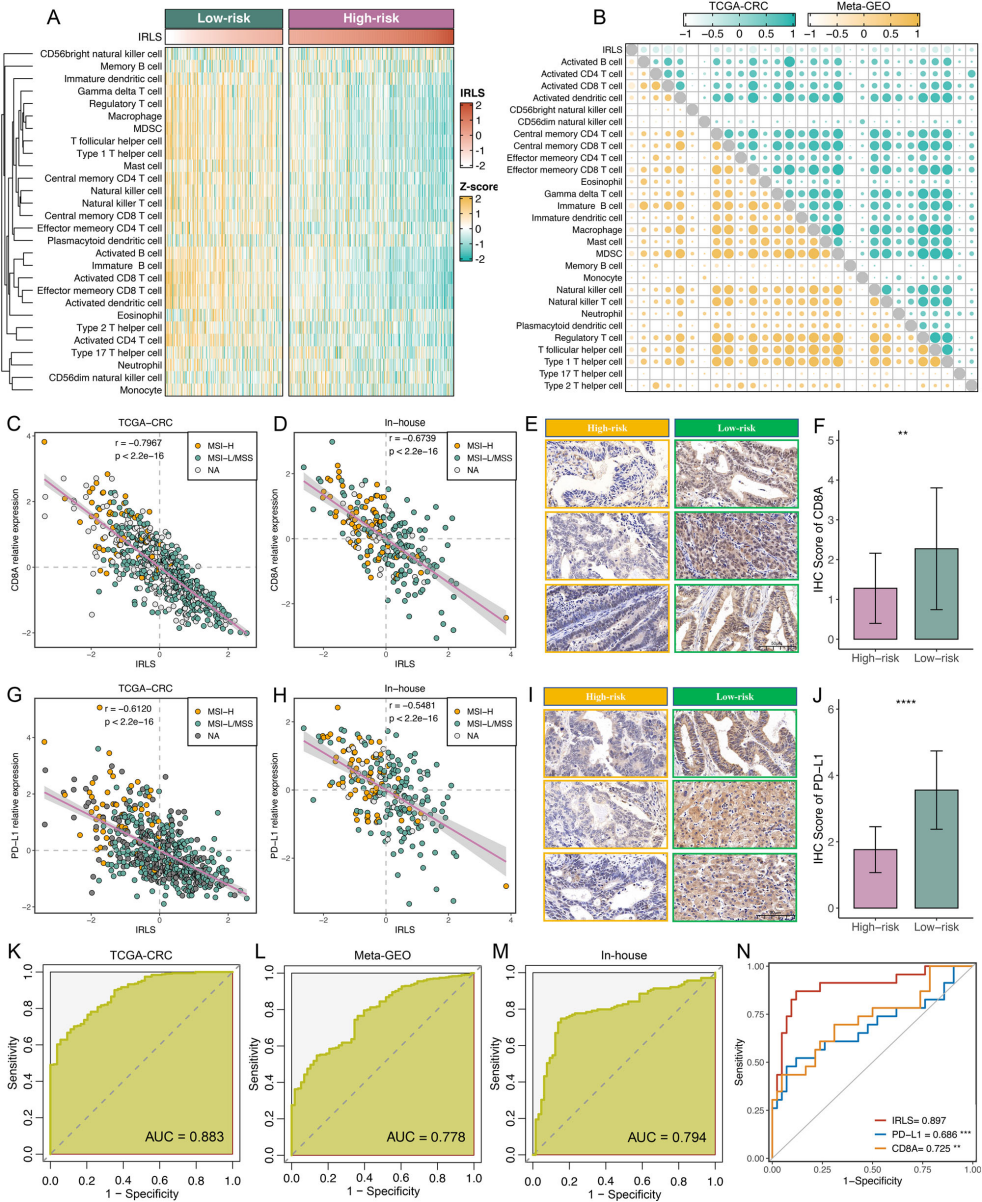
Implications of IRLS for ICI treatment. **A** The relationship between IRLS and immune cell infiltrations in TCGA-CRC. **B** Chorograms were derived based on Pearson r value between IRLS and immune cell infiltrations in TCGA-CRC and Meta-GEO. **C**, **D** Scatterplots between IRLS and CD8A expression with microsatellite state were shown in TCGA-CRC (*n* = 584, *P* = 5.20e−15) (**C**) and in-house cohort (*n* = 232, *P* = 4.45e−32) (**D**). Statistic test: Pearson’s correlation coefficient, two-sided unpaired *t* test. Data are presented as mean ± 95% confidence interval [CI]. **E** Representative IHC staining images of CD8A betwee*n* two risk groups (*n* = 104). Scale bars = 50 μm. **F** Analysis of IHC scores between two risk groups according to CD8A staining results (*n* = 104, *P* = 0.009). Statistic test: two-sided unpaired *t* test. Data are presented as mean ± 95% CI. **G**, **H**. Scatterplots between IRLS and PD-L1 expression with microsatellite state were shown in TCGA-CRC (*n* = 584, *P* = 1.30e−30) (**G**) and in-house cohort (*n* = 232, *P* = 1.37e−19) (**H**). Statistic test: Pearson’s correlation coefficient, two-sided unpaired *t* test. Data are presented as mean ± 95% CI. **I** Representative IHC staining images of PD-L1 betwee*n* two risk groups (*n* = 104). Scale bars = 50 μm. **J** Analysis of IHC scores between two risk groups according to PD-L1 staining results (*n* = 104, *P* = 1.34e−5). Statistic test: two-sided unpaired *t* test. Data are presented as mean ± 95% CI. **K**–**M** ROC curves of IRLS to predict the dMMR/MSI-H phenotype in TCGA-CRC (**K**), Meta-GEO (**L**), and in-house cohort (**M**). **N** ROC curves of IRLS, PD-L1, and CD8A to predict the benefits of pembrolizumab. Statistic test: two-sided unpaired DeLong test. **P < 0.01; ***P < 0.001; ****P < 0.0001.

Since the development of IRLS is based on immune-related lncRNAs, we assumed that there were differences in immune characteristics and immunotherapy effects at different levels of IRLS. Cell infiltration analysis showed a dramatically inverse correlation between IRLS and immune infiltrate abundance in both the TCGA-CRC and Meta-GEO cohorts (Fig. 7A, B and Supplementary Fig. 8A). Likewise, scatter plots of IRLS and *CD8A* demonstrated a negative correlation in the TCGA-CRC (*r* = −0.797, Fig. 7C), Meta-GEO (*r* = −0.711, Supplementary Fig. 8B), and in-house cohorts (*r* = −0.674, Fig. 7D). To further verify the protein expression of CD8A at different levels of IRLS, we performed IHC on paraffin sections, which included 56 high-risk CRC and 48 low-risk CRC samples. IHC images and scores displayed that the expression of CD8A was dramatically higher in the low-risk group (Fig. 7E, F). This indicated that patients with low IRLS possessed potentially more backup resources for ICI treatment. Additionally, IRLS was also negatively related to PD-L1 expression in the TCGA-CRC (*r* = −0.612, Fig. 7G), Meta-GEO (*r* = −0.389, Supplementary Fig. 8C), and in-house cohorts (*r* = −0.548, Fig. 7H). This consistent finding was also found at the protein level (Fig. 7I, J). Overall, IRLS was lower as CD8A and PD-L1 expression increased in the three cohorts (Supplementary Fig. 8D–F). In addition, IRLS demonstrated a predominant association with genomic instability, such as TMB (*r* = −0.218) and NAL (*r* = −0.222) (Supplementary Fig. 8G, H). The microsatellite state is also considered to be a strong biomarker for immune infiltration and ICI treatment in CRC^31^. In this study, we observed that patients with dMMR/MSI-H displayed significantly lower IRLS than those with pMMR/MSI-L/MSS (Supplementary Fig. 9). Of note, IRLS could accurately predict the dMMR/MSI-H phenotype in TCGC-CRC (AUC = 0.883), Meta-GEO (AUC = 0.778), and in-house cohorts (AUC = 0.794) (Fig. 7K–M), which suggested that IRLS is a favourable surrogate for microsatellite state estimation. In addition, we investigated the associations between IRLS and consensus molecular subtypes (CMS1-4). As illustrated in Supplementary Fig. 10A, the CMS1 subtype displayed a lower IRLS score than the other subtypes. As is well known, CMS1 belongs to the immune subtype, with a high fraction of MSI-H patients and better prognosis, in line with the indications of IRLS. In addition, we plotted ROC curves to further evaluate the accuracy of IRLS in the identification of CMS1 CRC patients, and the AUCs for IRLS were relatively high, at 0.915 (TCGA-CRC) and 0.859 (Meta-GEO) (Supplementary Fig. 10B). Subsequently, we further investigated the distribution of IRLS in 65 patients treated with pembrolizumab, of which 23 patients were included in the responder group (CR, *n* = 7; PR, *n* = 16) and 42 patients in the nonresponder group (SD, *n* = 18; PD, *n* = 24). As illustrated in Supplementary Fig. 11, responders displayed a lower level of IRLS than nonresponders. ROC analysis showed that IRLS could also markedly discriminate responders from nonresponders of pembrolizumab (AUC = 0.897) and was significantly superior to PD-L1 (AUC = 0.686, *P* < 0.001) and CD8A (AUC = 0.725, *P* < 0.01) expression (Fig. 7N).

## Discussion

The AJCC staging system is a conventional approach for clinical management such as treatment decision-making and surveillance strategies of CRC, but it is limited by heterogeneous clinical outcomes within the same stage. This insufficient approach might lead to underlying overtreatment or undertreatment^8^. With advancements in molecular biology and immunology, treatment modalities for CRC have also become diversified, for instance, antiangiogenic drugs (e.g. bevacizumab) and ICI treatment (e.g. nivolumab, ipilimumab)^32,33^. Diverse treatment options mean that patients need better personalised assessment ways to implement clinical decisions. However, reliable prognostic biomarkers that can identify “high-risk” CRC patients, who might benefit from ACT, bevacizumab, and ICI therapy are currently lacking^2^. To bridge this gap, we investigated the relationship between immune-related lncRNA profiles and prognosis, recurrence, and drug benefits.

In this study, two algorithms, WGCNA combined with consensus clustering and ImmLnc based on GSEA, were applied to identify immune-related lncRNAs. With the expression profiles of these lncRNAs, we developed an integrative pipeline to construct a consensus IRLS. In total, 101 kinds of models were fitted to the training dataset via the LOOCV framework. Further validations in six independent datasets revealed that the optimal model was a combination of Lasso and stepwise Cox (direction = both). The advantage of integrative procedures is to fit a model with consensus performance on the prognosis of CRC based on a variety of machine learning algorithms and their combinations, and algorithm combinations can further reduce the dimensionality of variables, making the model more simplified and translational. The prognostic meta-analysis demonstrated that IRLS was a deleterious indicator of OS and RFS, and was proven to be an independent factor for OS rather than RFS. Thus, IRLS is more suitable for evaluating OS in CRC, but has limited predictive value for RFS. In addition, ROC and C-index analysis suggested that IRLS maintained the high accuracy and stable performance in seven public datasets and an in-house cohort, which indicated great potential for the clinical application of IRLS.

The T, N, M, and AJCC stages are conventional tools for evaluating clinical outcomes and treatment decisions^3^. Additionally, whether to use ACT and emerging biomarkers, including TMB; NAL; microsatellite state; and TP53, KRAS, or BRAF mutations, are also significantly correlated with the clinical strategies and outcomes^24,34^. Notably, our signature worked independently of these factors and also had significantly superior performance in predicting prognosis according to the C-index assessment. In addition, we retrieved 109 published signatures containing various functional gene combinations. Among these signatures, few have been incorporated into clinical practice, and even fewer have been thoroughly validated^2^. For example, univariate Cox regression displayed that, except for IRLS, no signature maintained prognostic significance across all cohorts. With the comparison of predictive superiority among these signatures, IRLS also presented better performance in every dataset than almost all models. We noticed that most models performed well in their own training dataset and a few external datasets (e.g. Chen-Gene, Dai-FIG) but performed weakly in other datasets^26,27^. This may be due to the poor generalisability of the model derived by overfitting. Our signature was reduced dimensionally by two machine learning algorithms and therefore had a better extrapolation possibility. To further test the clinical interpretation of IRLS, another validation was based on qRT-PCR results from 232 frozen CRC tissues, verifying our prior findings and assessing their feasibility in different centres. Therefore, our signature could be a promising surrogate for evaluating the prognosis of CRC in clinical settings.

Fluorouracil-based ACT (FOLFOX or FOLFIRI) in CRC is the standard modality in stage III but remains controversial in stage II^3^. Current prognostic markers utilised in clinical practice are inadequate to identify patients with stage II CRC at high risk of recurrence or patients with stage III CRC at low risk, hence giving rise to latent overtreatment or undertreatment with ACT^8^. Moreover, several studies have demonstrated that fluorouracil-based ACT in combination with bevacizumab can extend OS in CRC patients relative to those receiving fluorouracil-based ACT alone^35,36^. Nevertheless, bevacizumab benefits only a subset of patients, and it can lead to high costs and serious side effects. With the objective of improving this clinical conundrum, we investigated the predictive value of IRLS for measuring the benefits of ACT and bevacizumab. Indeed, accumulating evidence has demonstrated that lncRNAs are closely associated with the responses to ACT and bevacizumab^18,28–30^. In this study, we found that patients with high IRLS were sensitive to fluorouracil-based ACT alone, while patients with low IRLS were more prone to respond to fluorouracil-based ACT in combination with bevacizumab. ROC analysis indicated that IRLS afforded greater accuracy in the prediction of fluorouracil-based ACT and bevacizumab benefits. Thus, the IRLS system might be a powerful tool for tailoring decision-making for CRC patients.

Cancer immunotherapy represented by ICIs has revolutionised the treatment of solid tumours, including a subset of CRC. Two monoclonal antibodies targeting PD-1, nivolumab and pembrolizumab, have demonstrated considerable benefits in CRC with MSI-H or dMMR^37^. In this study, patients with low IRLS displayed higher TMB and NAL. TMB could increase the production of mutation-derived neoantigens and enhance tumour immunogenicity, which further induces the proliferation and activation of cytotoxic T lymphocytes^38^. Actually, patients with low IRLS presented abundant immune cell infiltration, indicating an “immune-hot” phenotype. CD8A and PD-L1 also showed a high distribution of both RNA and protein in patients with low IRLS. These results suggested that the low level of IRLS indicates more backup lymphocyte resources and potentially greater sensitivity to ICI treatment. Meanwhile, patients with dMMR/MSI-H were prone to have a higher distribution of IRLS, which was consistent with previously reported dMMR/MSI-H tumours having better prognosis and more tumour-infiltrating lymphocytes^37^. However, the dMMR/MSI-H phenotype only accounts for less than 5% of tumours, hindering its clinical utilisation^7^. Additionally, IRLS could accurately predict the dMMR/MSI-H phenotype in three cohorts, which suggested that IRLS is a favourable surrogate for microsatellite state estimation. Further in-house estimation indicated that IRLS could markedly discriminate responders from nonresponders to pembrolizumab, significantly better than two well-studied biomarkers, PD-L1 and CD8A. Therefore, IRLS is also a candidate biomarker for assessing the benefits of ICI treatment, and patients with high IRLS might not be suitable for ICI treatment due to potential resistance and immune-related adverse events (irAEs).

The IRLS model can be reproduced using a simple PCR-based assay, making it attractive for clinical translation and implementation. Although the clinical significance of IRLS in CRC is promising, some limitations should be acknowledged. First, all of the samples from this study were retrospective, and future validation of IRLS should be performed in a prospective multicentre cohort. Second, some clinical and molecular traits on public datasets were very inadequate, which may have concealed the potential associations between IRLS and certain variables. Third, the roles of most lncRNAs from IRLS in CRC remain unknown, and further in vivo and in vitro experiments are needed to reveal their functions.

In conclusion, based on a multitude of bioinformatics and machine learning algorithms, we developed a stable and powerful signature for assessing the prognosis, recurrence, and benefits of fluorouracil-based ACT, bevacizumab, and pembrolizumab. This IRLS model is a promising tool to optimise decision-making and surveillance protocols for individual CRC patients.

## Methods

### Publicly available data collection and processing

In total, 2277 CRC patients from 17 independent public datasets were accessed from The Cancer Genome Atlas (TCGA) and Gene Expression Omnibus (GEO) (Supplementary Data 5). Among these, seven datasets (TCGA-CRC, GSE17536, GSE17537, GSE29621, GSE38832, GSE39582, and GSE72970) encompassing complete OS and RFS information were used for the construction and validation of our signature. Four datasets (GSE31595, GSE92921, GSE143985, and GSE161158) containing only RFS information were used to verify the predictive value of IRLS for recurrence. For drug-related datasets, we enroled six datasets treated with fluorouracil-based ACT (FOLFOX or FOLFIRI) alone: GSE19860, GSE28702, GSE45404, GSE62080, GSE69657, and GSE72970, which included 180 nonresponders and 160 responders. In addition, three datasets (GSE19860, GSE19862, and GSE72970), including 30 nonresponders and 24 responders of fluorouracil-based ACT in combination with bevacizumab, were also collected. These drug-related datasets were applied to assess the performance of IRLS in predicting ACT and bevacizumab benefits in CRC.

The RNA-seq raw read count from the TCGA database was converted to transcripts per kilobase million (TPM) and further log-2 transformed. Data from the GEO database were all retrieved from the Affymetrix^®^ GPL570 platform (Human Genome U133 Plus 2.0 Array). The raw data from Affymetrix^®^ were processed via the robust multiarray averaging (RMA) algorithm implemented in the *Affy* package. According to the gene annotations in GENCODE (Homo sapiens GRCh38), 15299 lncRNA and 19526 protein-coding genes were included in the TCGA datasets. We reannotated probe sets of the GPL570 array for genes by mapping all probes to the human genome (hg38) using SeqMap^39^ and then obtained 3439 lncRNA and 17046 protein-coding genes. After removing batch effects by the *ComBat* algorithm, the TCGA-CRC cohort was combined from the TCGA-COAD and TCGA-READ datasets, and the Meta-GEO cohort was combined from all GEO datasets belonging to the Affymetrix^®^ GPL570 platform. Each gene expression was transformed into z-score across patients in all cohorts. The detailed baselines of the 17 enroled datasets are summarised in Supplementary Data 5.

### Cells infiltration estimation

Single-sample gene set enrichment analysis (ssGSEA) implemented in R package *GSVA* was employed to quantify the relative infiltration of 28 immune cells in the TCGA-CRC cohort^21^. Six other algorithms including TIMER, quanTIseq, MCP-counter, xCell, EPIC, and ESTIMATE, were further performed to verify the stability and robustness of the ssGSEA results.

### Consensus clustering

According to the infiltration profile of various immune cells, a resampling-based method termed consensus clustering was applied for cluster discovery in the TCGA-CRC cohort^22^. This process was performed by the *ConsensusClusterPlus* package. Subsequently, the consensus score matrix, CDF curve, PAC score, and Nbclust were synthetically used to determine the optimal number of clusters^23^. See Supplementary Information for details.

### Weighted correlation network analysis (WGCNA)

Coexpression lncRNA networks of TCGA-CRC were generated using the *WGCNA* package. An appropriate soft threshold β was calculated to meet the criteria for the scale-free network. Furthermore, the weighted adjacency matrix was converted into a topological overlap matrix (TOM), and the corresponding dissimilarity was generated (1-TOM). The dynamic tree cutting approach was employed to conduct the module identification. To recognise lncRNA modules significantly correlated with immune clusters, the module that displayed the highest correlation was selected for further study. lncRNAs with both high GS and MM were defined as immune-related lncRNAs.

### ImmLnc analysis framework

ImmLnc is an integrated algorithm for identifying lncRNA modulators of immune-related pathways. First, the ESTIMATE algorithm was used to infer tumour purity. Second, we calculated the partial correlation coefficient (PCC) between a specific lncRNA and all mRNAs by adjusting the tumour purity as a covariable. Finally, all mRNAs were ranked by the correlation coefficient with a specific lncRNA, and the ranked gene list was further subjected to GSEA procedure to investigate whether the immune genes were enriched in the top or bottom of the gene list. As recommended, lncRES scores >0.995 and FDR < 0.05 were considered statistically significant^9,18^.

### Signature generated from machine learning-based integrative approaches

To develop a consensus IRLS with high accuracy and stability performance, we integrated 10 machine learning algorithms and 101 algorithm combinations. The integrative algorithms included random survival forest (RSF), elastic network (Enet), Lasso, Ridge, stepwise Cox, CoxBoost, partial least squares regression for Cox (plsRcox), supervised principal components (SuperPC), generalised boosted regression modelling (GBM), and survival support vector machine (survival-SVM). The signature generation procedure was as follows: (a) Univariate Cox regression identified prognostic lncRNAs in the TCGA-CRC cohort; (b) Then, 101 algorithm combinations were performed on the prognostic lncRNAs to fit prediction models based on the leave-one-out cross-validation (LOOCV) framework in the TCGA-CRC cohort; (c) All models were detected in six validation datasets (GSE17536, GSE17537, GSE29621, GSE38832, GSE39582, and GSE72970); (d) For each model, the Harrell’s concordance index (C-index) was calculated across all validation datasets, and the model with the highest average C-index was considered optimal. See Supplementary Information for details.

### Human tissue specimens and quantitative real-time PCR (qRT-PCR)

The human cancer tissues used in this study were approved by Ethnics Committee of The First Affiliated Hospital of Zhengzhou University on December 19, 2019, and the TRN is 2019-KW-423. Overall, 232 frozen surgically resected CRC tissues were collected from The First Affiliated Hospital of Zhengzhou University. All patients provided written informed consent; received available standard systemic therapies (fluorouracil, oxaliplatin, irinotecan, and pembrolizumab); were aged 18 years or older; had adequate haematologic, renal, and liver function; had Eastern Cooperative Oncology Group performance status of 0 or 1; and had measurable disease according to Response Evaluation Criteria in Solid Tumours (RECIST, version 1.1)^40^. Responders and nonresponders were defined as having a complete response (CR)/partial response (PR) and stable disease (SD)/progressive disease (PD), respectively. Detailed baseline data of CRC patients are displayed in Supplementary Data 5. Total RNA was isolated from CRC tissues using RNAiso Plus reagent RNA quality was evaluated using a NanoDrop One C (Waltham, MA, USA), and RNA integrity was assessed using agarose gel electrophoresis. The primer sequences of the 16 lncRNAs and GAPDH are shown in Supplementary Data 6. See Supplementary Information for details.

### Immunohistochemistry (IHC)

For the IHC assay, paraffin sections were incubated with primary antibodies against CD8A (1:300; Cat# GB13068-2; Servicebio, Wuhan, China) and PD-L1 (1:500; Cat# GB11339; Servicebio, Wuhan, China) at 37 °C for 60 min, secondary antibodies at 37 °C for 15 min and horseradish enzyme-labelled streptavidin solution for 10 min and then stained with DAB and haematoxylin. Staining percentage scores were classified as follows: 1 (1–25%), 2 (26–50%), 3 (51–75%) and 4 (76–100%), and staining intensity was scored 0 (signalless colour) to 3 (light yellow, brown, and dark brown). The stained tissues were scored by three individuals blinded to the clinical parameters. A final IHC score was calculated by multiplying the scores of “percentage of protein-positive cells” and “intensity of nuclear staining”.

### Statistical analysis

All data processing, statistical analysis, and plotting were conducted in R 4.0.5 software. Correlations between two continuous variables were assessed via Pearson’s correlation coefficients. The chi-squared test was applied to compare categorical variables, and continuous variables were compared through the Wilcoxon rank-sum test or T test. The *survminer* package was used to determine the optimal cut-off value. Cox regression and Kaplan–Meier analyses were performed via the *survival* package. The C-indices of different variables were compared using the *CompareC* package. The receiver operating characteristic curve (ROC) used to predict binary categorical variables was implemented via the *pROC* package. The time-dependent area under the ROC curve (AUC) for survival variables was conducted by the *timeROC* package. The iAUC was generated by the *risksetROC* package and the IBS was calculated using the *survcomp* package. The CMS subtypes were inferred via the *CMSclassifier* package^41^. All statistical tests were two-sided. *P* < 0.05 was regarded as statistically significant.

### Reporting summary

Further information on research design is available in the Nature Research Reporting Summary linked to this article.

## Supplementary information

Supplementary Information

Description of Additional Supplementary Files

Supplementary Data 1

Supplementary Data 2

Supplementary Data 3

Supplementary Data 4

Supplementary Data 5

Supplementary Data 6

Reporting Summary

## Data Availability

Public data used in this work can be acquired from the TCGA Research Network portal (https://portal.gdc.cancer.gov/) and Gene Expression Omnibus (GEO, http://www.ncbi.nlm.nih.gov/geo/).

## Code availability

Essential scripts for implementing machine learning-based integrative procedure in multiple independent datasets are available on the Github website (https://github.com/Zaoqu-Liu/IRLS).
