# Supplementary material for: Machine learning-based integration develops an immune-derived lncRNA signature for improving outcomes in colorectal cancer
Source: Nat Commun. 2022 Feb 10;13:816. doi: 10.1038/s41467-022-28421-6 (PMC8831564; doi:10.1038/s41467-022-28421-6)
Supplement: Supplementary file 1 — Supplementary Information [file 41467_2022_28421_MOESM1_ESM.pdf]

Supplementary Information

- Supplementary Figure 1
- Supplementary Figure 2
- Supplementary Figure 3
- Supplementary Figure 4
- Supplementary Figure 5
- Supplementary Figure 6
- Supplementary Figure 7
- Supplementary Figure 8
- Supplementary Figure 9
- Supplementary Figure 10
- Supplementary Figure 11
- Supplementary Methods

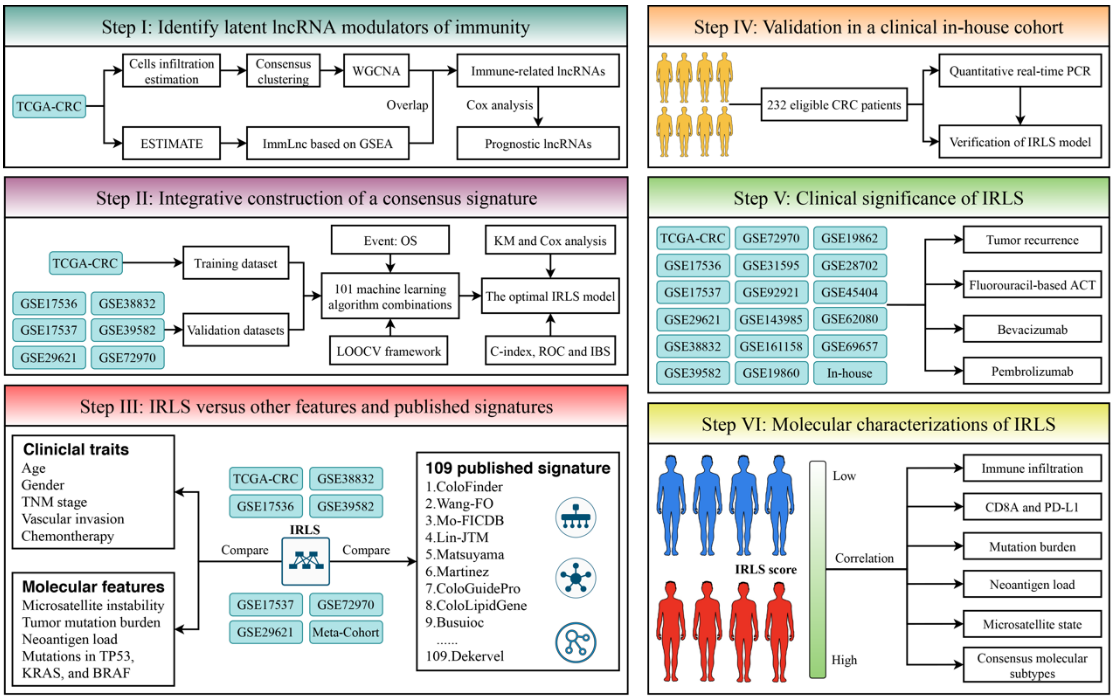

Supplementary Figure 1. The overall design of this study. The human figure outline was sourced from the free website smart server medical art (https://smart.servier.com/).

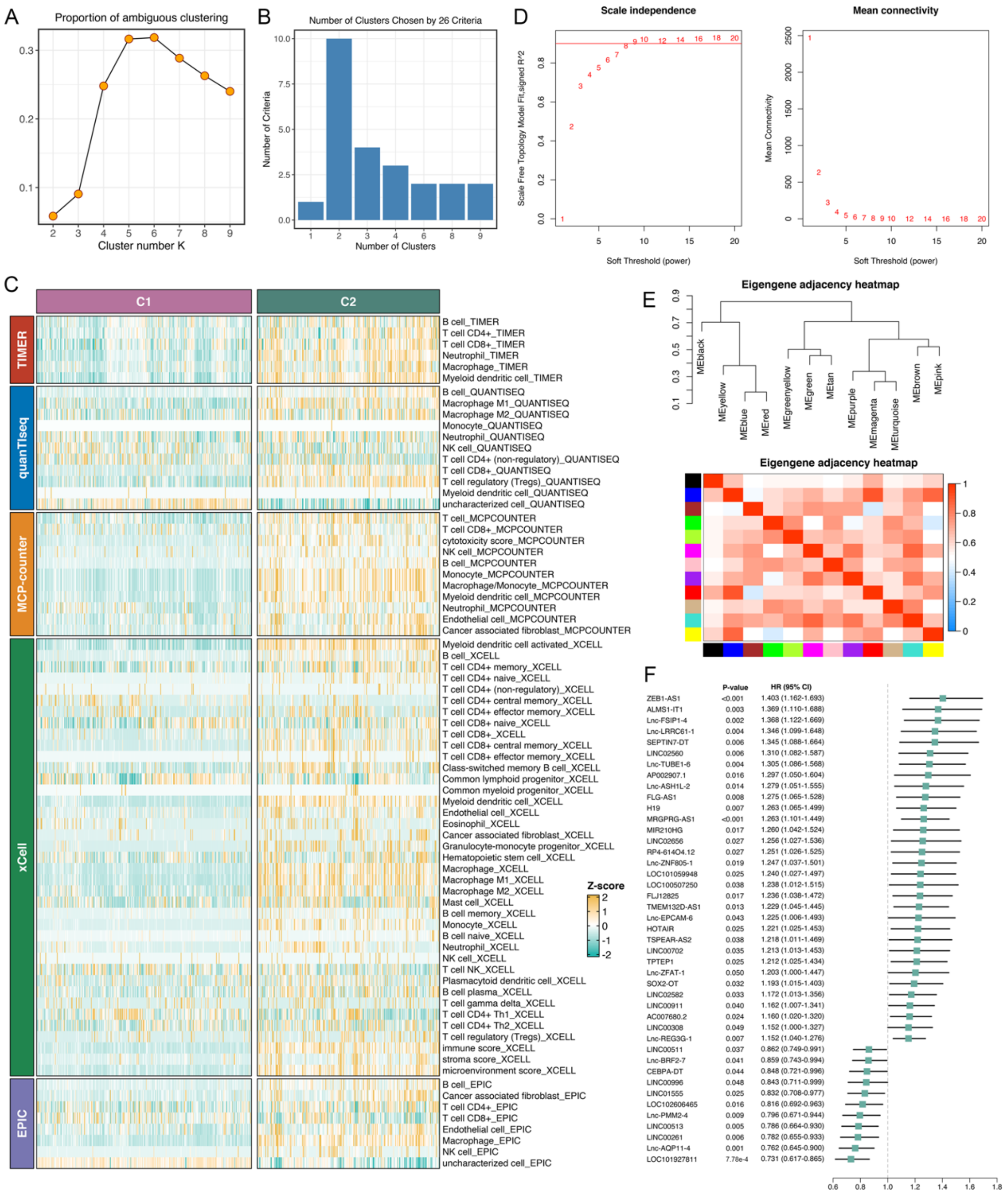

Supplementary Figure 2. Consensus clustering and WGCNA analysis. A. The proportion of ambiguous clustering (PAC) score, a low value of PAC implies a flat middle segment, allowing conjecture of the optimal k (k = 2) by the lowest PAC. **B.** Recommended number of clusters using 26 criteria of Nbclust package. **C**. Five other algorithms including TIMER, quanTIseq, MCP-counter, xCell, and EPIC, further verified the stability and robustness of the ssGSEA results. **D**. Analysis of network topology for different soft-threshold power. The left panel shows the impact of soft-threshold power on the scale-free topology fit index; the right panel displays the impact

of soft-threshold power on the mean connectivity. **E**. The heatmap revealed the eigengene adjacency of modules. **F**. Univariate Cox analysis identified 43 prognostic lncRNAs in the TCGA-CRC cohort (n =584). Statistic tests: two-sided Wald test. Data are presented as hazard ratio (HR) ± 95% confidence interval [CI].

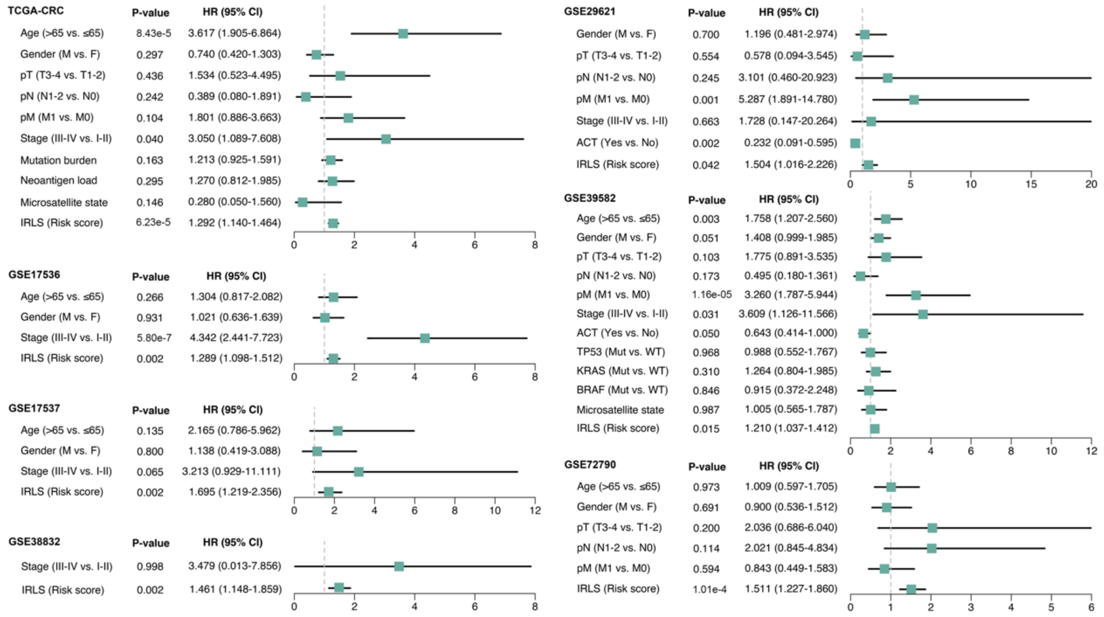

Supplementary Figure 3. Multivariate Cox regression of IRLS regarding to OS in TCGA-CRC (n =584), GSE17536 (n =177), GSE17537 (n =55), GSE29621 (n =65), GSE38832 (n =122), GSE39582 (n =573), and GSE72970 (n =124). Statistic tests: two-sided Wald test. Data are presented as hazard ratio (HR) ± 95% confidence interval [CI].

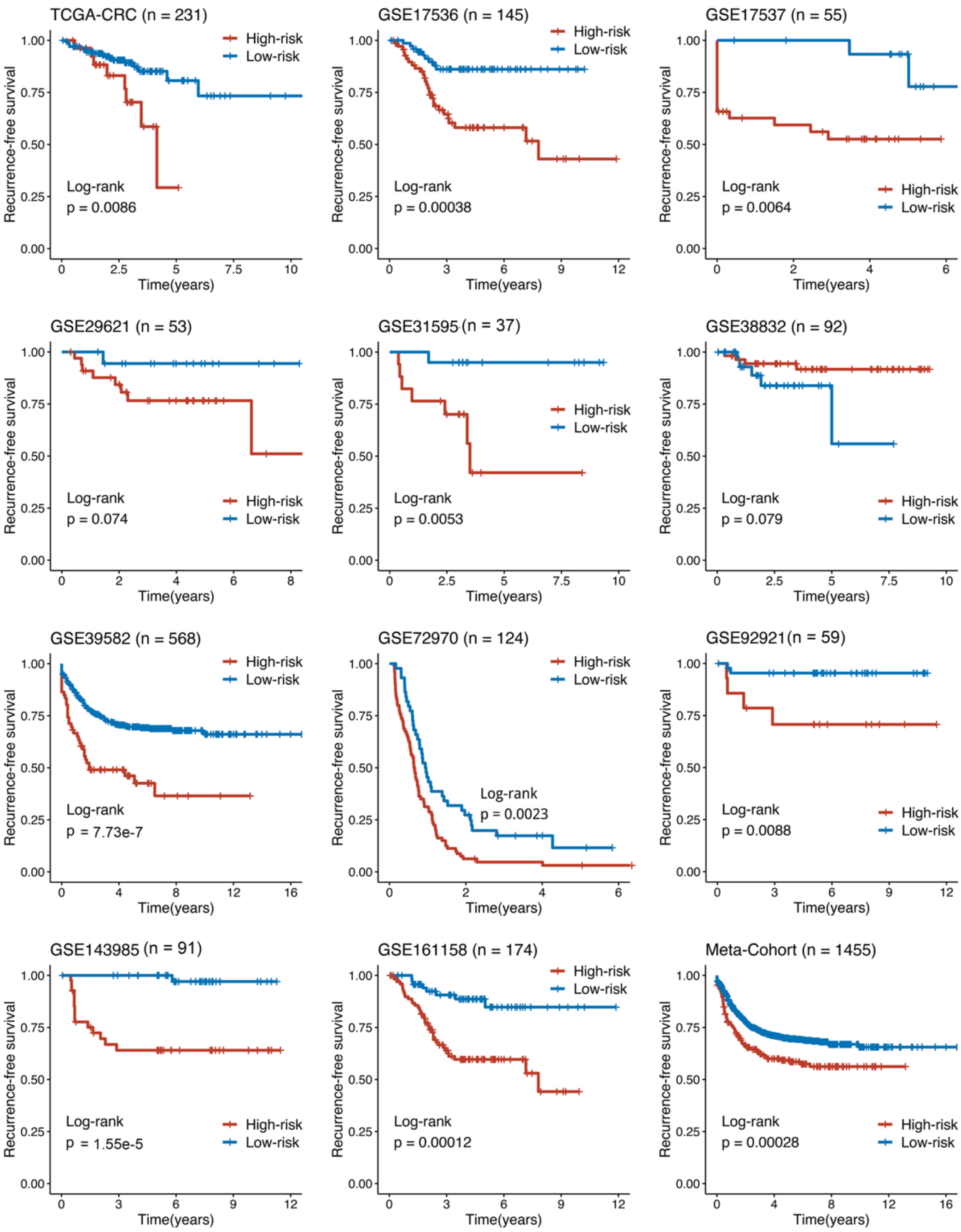

Supplementary Figure 4. Kaplan-Meier curves of RFS according to the IRLS in TCGA-CRC, GSE17536, GSE17537, GSE29621, GSE31595, GSE38832, GSE39582, GSE72970, GSE92921, GSE143985, GSE161158, and meta-cohort.

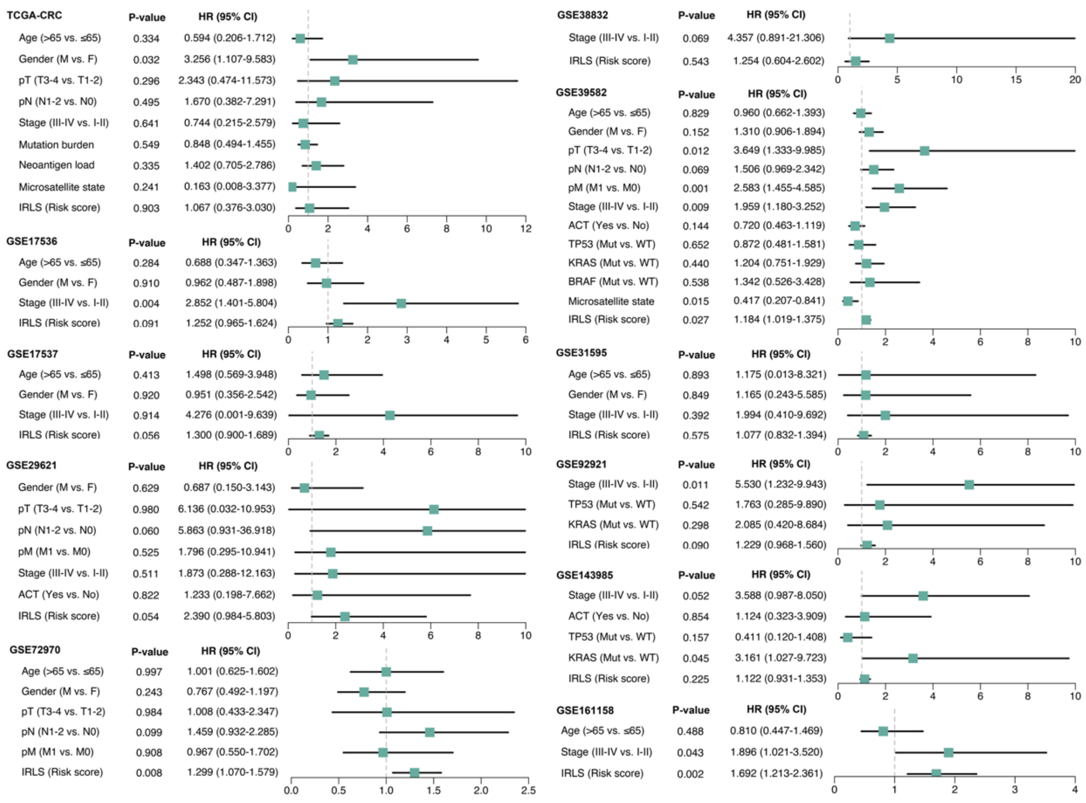

Supplementary Figure 5. Multivariate Cox regression of IRLS regarding to RFS in TCGA-CRC (n =231), GSE17536 (n =145), GSE17537 (n =55), GSE29621 (n =53), GSE31595 (n =37), GSE38832 (n =92), GSE39582 (n =568), GSE72970 (n =124), GSE92921 (n =59), GSE143985 (n =91), and GSE161158 (n =174). Statistic tests: two-sided Wald test. Data are presented as hazard ratio (HR) ± 95% confidence interval [CI].

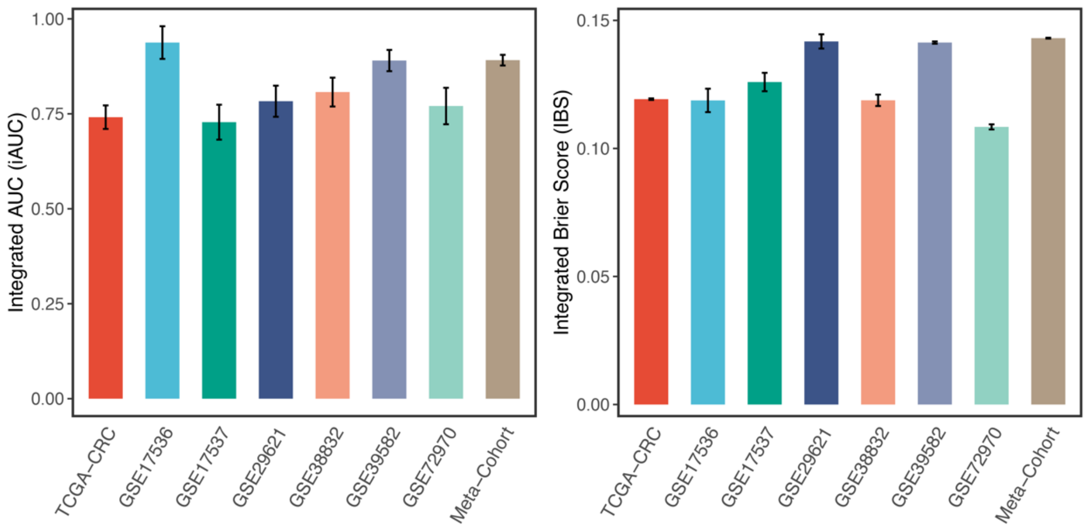

Supplementary Figure 6. The iAUC and IBS of IRLS in TCGA-CRC (n =584),

GSE17536 (n =177), GSE17537 (n =55), GSE29621 (n =65), GSE38832 (n =122), GSE39582 (n =573), GSE72970 (n =124), and meta-cohort (n =1700). Data are presented as mean ± 95% confidence interval [CI].

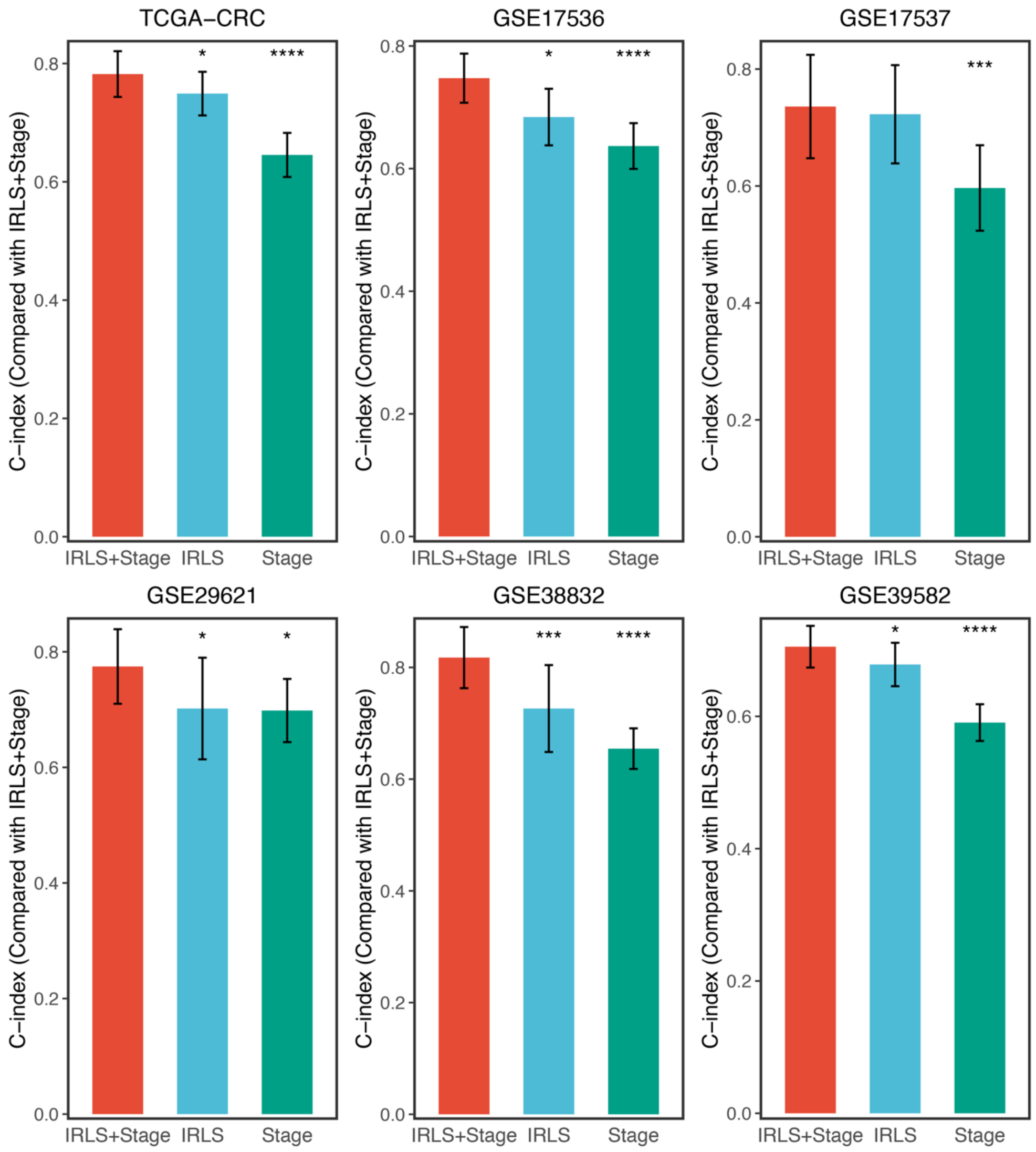

Supplementary Figure 7. The performance of IRLS+Stage was compared with IRLS and AJCC stage alone in predicting prognosis in TCGA-CRC (n =584), GSE17536 (n =177), GSE17537 (n =55), GSE29621 (n =65), GSE38832 (n =122), and GSE39582 (n =573). Statistic tests: two-sided z-score test. Data are presented as mean ± 95%

confidence interval [CI]. *P <0.05, ***P <0.001, ****P <0.0001.

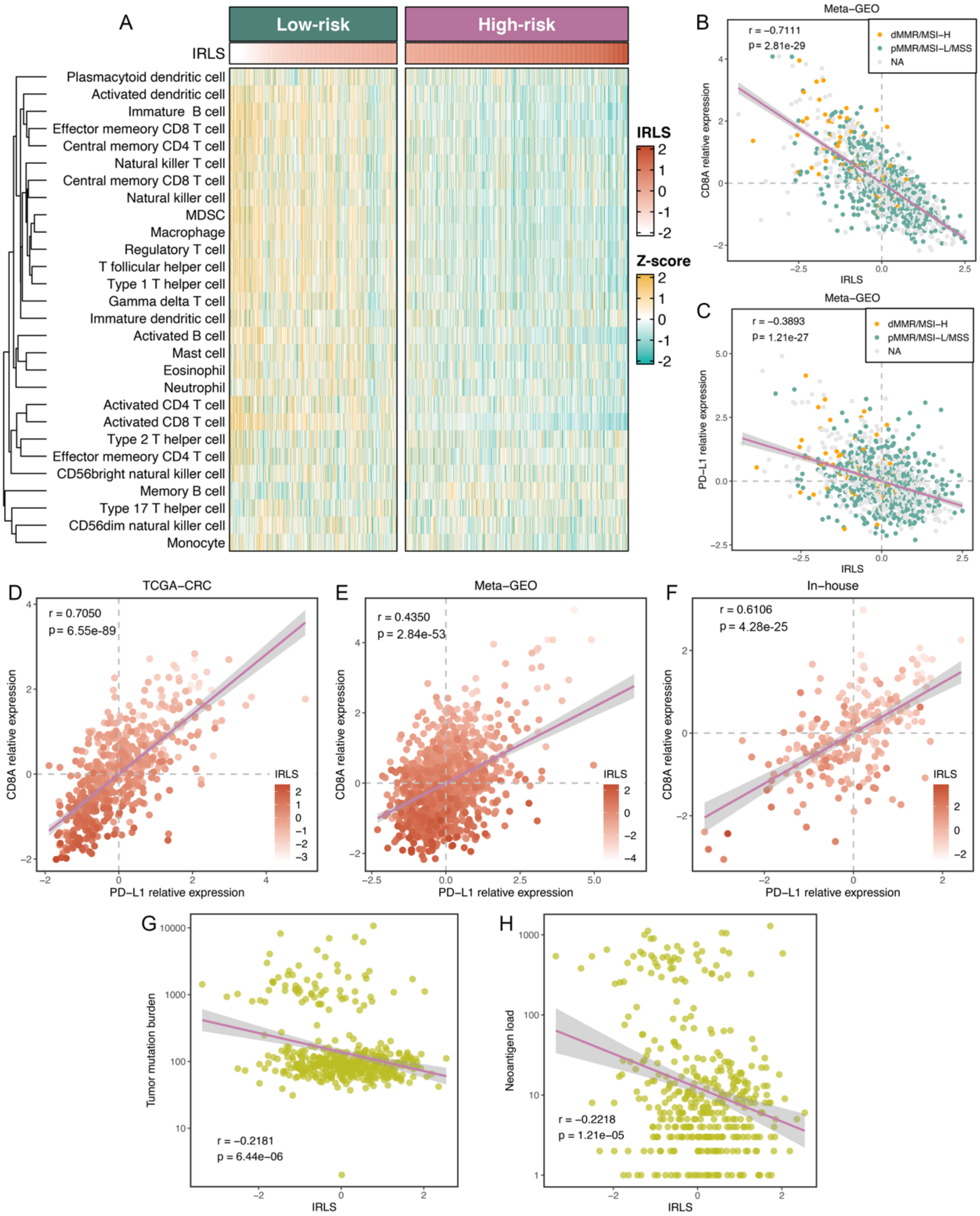

Supplementary Figure 8. Implications of IRLS for ICI treatment. A. The relationship between IRLS and immune cell infiltrations in Meta-GEO. **B-C**. Scatterplots between IRLS and CD8A (**B**) and PD-L1 (**C**) expression with microsatellite state were shown in Meta-GEO. **D-F**. Scatterplots between CD8A and PD-L1 expression with the IRLS score were shown in TCGA-CRC (**D**), Meta-GEO (**E**),

and in-house cohort (**F**). **G-H**. Scatterplots between IRLS and TMB (**G**) and NAL (**H**). Statistic tests: Pearson's correlation coefficient, two-sided unpaired t-test. Data in (**B-H**) are presented as mean ± 95% confidence interval [CI].

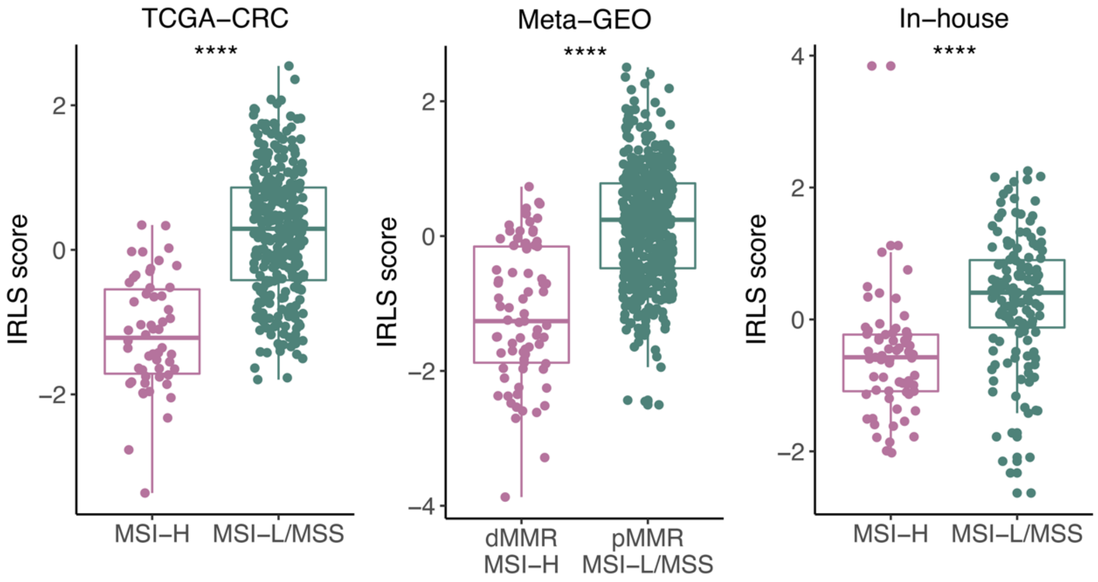

Supplementary Figure 9. Relationships between IRLS and microsatellite status in TCGA-CRC (n =366, *P* =1.03e-19), Meta-GEO (n =527, *P* =1.17e-10), and in-house (n =204, *P* =1.04e-9) cohorts. In boxplot graphs centre line indicates median, bounds of box indicate 25th and 75th percentiles, and whiskers indicate minimum and maximum. Statistic tests: two-sided unpaired t-test. ****$P < 0.0001$.

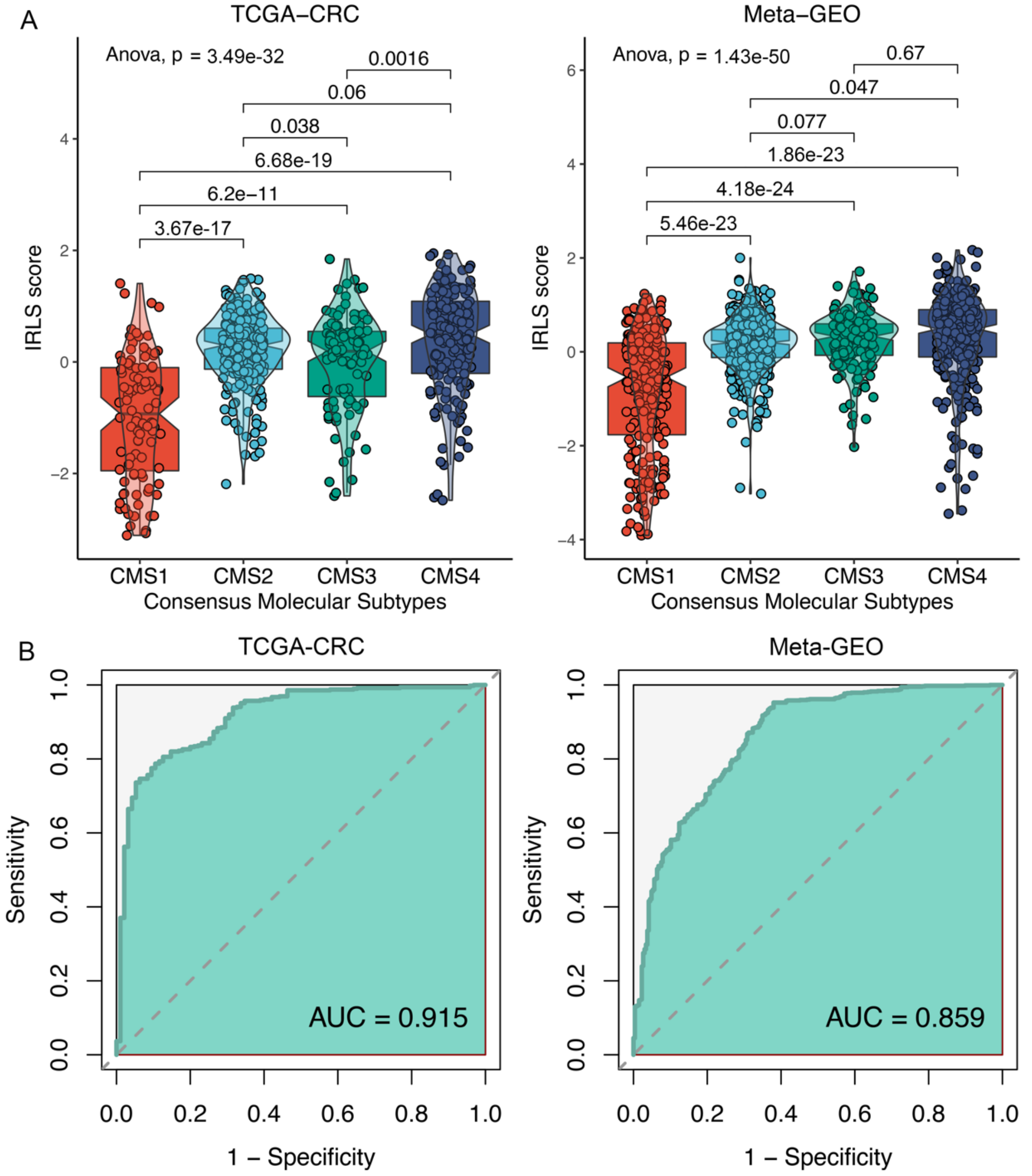

Supplementary Figure 10. Relationships between IRLS and consensus molecular subtypes. A. The distribution of IRLS score among CMS1-4 subtypes in TCGA-CRC (n =584) and Meta-GEO (n =1700) cohorts. In boxplot graphs centre line indicates median, bounds of box indicate 25th and 75th percentiles, and whiskers indicate minimum and maximum. **B**. ROC curves of IRLS to predict the CMS1 subtype in TCGA-CRC and Meta-GEO cohorts.

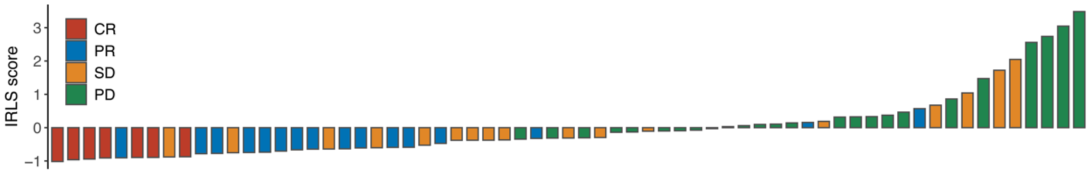

Supplementary Figure 11. Distributions of IRLS in different pembrolizumab response groups.

Supplementary Methods

Consensus clustering

According to the infiltration profile of various immune cells, a resampling-based method termed consensus clustering was applied for cluster discovery. This process was performed by *ConsensusClusterPlus* package[1]. Subsample 80% of samples at each iteration and partition each subsample into up to k (max K = 9) groups by k-means algorithm upon Euclidean distance. This process was repeated for 1,000 repetitions. Subsequently, the consensus score matrix, cumulative distribution function (CDF) curve, proportion of ambiguous clustering (PAC) score, and Nbclust were synthetically used to determine the optimal number of clusters. A higher consensus score between two samples indicates they are more likely to be grouped into the same cluster in different iterations. The consensus values range from 0 (never clustered together) to 1 (always clustered together) marked by white to dark brown. In the CDF curve of a consensus matrix, the lower left portion represents sample pairs rarely clustered together, the upper right portion represents those almost always clustered together, whereas the middle segment represents those with ambiguous assignments in different clustering runs. The "proportion of ambiguous clustering" (PAC) measure quantifies this middle segment; and is defined as the fraction of sample pairs with consensus indices falling in the interval $(u1, u2) \in [0, 1]$ where u1 is a value close to 0 and u2 is a value close to 1 (for instance u1=0.1 and u2=0.9). A low value of PAC indicates a flat middle segment, and a low rate of discordant assignments across permuted clustering runs. PAC for each K is $CDF_k(u2) - CDF_k(u1)$[2]. According to his criterion, we can therefore infer the optimal number of clusters by the K value having the lowest PAC.

The Nbclust uses 26 mathematic criteria to select the optimal number.

Signature generated from machine learning based integrative approaches

To develop a consensus immune-related lncRNA signature (IRLS) with high accuracy and stability performance, we integrated 10 machine learning algorithms including random survival forest (RSF), elastic network (Enet), Lasso, Ridge, stepwise Cox, CoxBoost, partial least squares regression for Cox (plsRcox), supervised principal components (SuperPC), generalized boosted regression modeling (GBM), and survival support vector machine (survival-SVM). A few algorithms possessed the ability of feature selection, such as Lasso, stepwise Cox, CoxBoost, and RSF. Thus, we combined these algorithms to generate a consensus model. In total, 101 algorithm combinations were conducted to fit prediction models based on the leave-one-out cross-validation (LOOCV) framework. The initial signature discovery was performed in TCGA-CRC. The RSF model was implemented via the *randomForestSRC* package. RSF had two parameters *ntree* and *mtry*, where *ntree* represented the number of trees in the forest and *mtry* was the number of randomly selected variables for splitting at each node. We used a grid-search on ntree and mtry using LOOCV framework. All the pairs of (*ntree*, *mtry*) are formed and the one with the best C-index value is identified as the optimized parameters. The Enet, Lasso, and Ridge were implemented via the *glmnet* package. The regularization parameter, λ, was determined by LOOCV, whereas the L1-L2 trade-off parameter, α, was set to 0-1 (interval =0.1). The stepwise Cox model was implemented via s*urvival* package. A stepwise algorithm using the AIC (Akaike information criterion) was applied, and the direction mode of stepwise search was set to "both", "backward", and "forward", respectively. The CoxBoost model was implemented via *CoxBoost* package, which is used to fit a Cox proportional hazards model by componentwise likelihood-based boosting. For the CoxBoost model, we used LOOCV routine *optimCoxBoostPenalty* function to first determine the optimal penalty (amount of shrinkage). Once this parameter was determined, the other tuning parameter of the algorithm, namely, the number of boosting steps to perform, was selected via the function *cv.CoxBoost*. The dimension of the selected multivariate Cox model was

finally set by the principal routine CoxBoost. The plsRcox model was implemented via *plsRcox* package. The *cv.plsRcox* function was used to determine the number of components requested, and the *plsRcox* function was applied to fit a partial least squares regression generalized linear model. The SuperPC model was implemented via *superpc* package, is a generalization of principal component analysis, which generates a linear combination of the features or variables of interest that capture the directions of largest variation in a dataset. The superpc.cv function used a form of LOOCV to estimate the optimal feature threshold in supervised principal components. To avoid problems with fitting Cox models to small validation datasets, it uses the "pre-validation" approach. The GBM model was implemented via *superpc* package. Using the LOOCV, the *cv.gbm* function selected index for number trees with minimum cross-validation error. The *gbm* function was used to fit the generalized boosted regression model. The survival-SVM model was implemented via *survivalsvm* package. The regression approach takes censoring into account when formulating the inequality constraints of the support vector problem.

RNA preparation and Quantitative Real-Time PCR (qRT-PCR)

Total RNA was isolated from CRC tissues using RNAiso Plus reagent RNA quality was evaluated using a NanoDrop One C (Waltham, MA, USA), and RNA integrity was assessed using agarose gel electrophoresis. An aliquot of 1 μg of total RNA was reverse transcribed into complementary DNA (cDNA) according to the manufacturer's protocol using a High-Capacity cDNA Reverse Transcription kit (TaKaRa BIO, Japan). qRT-PCR was performed using SYBR Assay I Low ROX (Eurogentec, USA) and SYBR® Green PCR Master Mix (Yeason, Shanghai, China) to detect the expression of 16 lncRNAs expression. The expression value was normalized to *GAPDH*, and then log2 transformed for subsequent analysis. The primer sequences of the included 16 lncRNAs and *GAPDH* were shown in Supplementary Data 6.

Supplementary References

1 Wilkerson, M. D. & Hayes, D. N. ConsensusClusterPlus: a class discovery tool

with confidence assessments and item tracking. *Bioinformatics* **26**, 1572-1573 (2010).

2 Şenbabaoğlu, Y., Michailidis, G. & Li, J. Z. Critical limitations of consensus clustering in class discovery. *Sci Rep-Uk* **4**, 6207-6207 (2014).
