# Supplementary material for: Machine learning-based integration develops an immune-derived lncRNA signature for improving outcomes in colorectal cancer
Source: Nat Commun. 2022 Feb 10;13:816. doi: 10.1038/s41467-022-28421-6 (PMC8831564; doi:10.1038/s41467-022-28421-6)
Supplement: Supplementary file 2 — Description of Additional Supplementary Files [file 41467_2022_28421_MOESM2_ESM.pdf]

Description of Additional Supplementary Files

Supplementary Data 1. A total of 791 lncRNAs was identified via ImmLnc algorithm.

Supplementary Data 2. The performance of 101 predictive models in training and testing cohorts.

Supplementary Data 3. The evaluation metrics of IRLS in training and testing cohorts.

Supplementary Data 4. A total of 109 published signatures were retrieved from the literatures.

Supplementary Data 5. Details of baseline information in 17 public datasets and an in-house dataset.

Supplementary Data 6. The forward and reverse primers for qRT-PCR.
