# Supplementary material for: Machine learning-based integration develops an immune-derived lncRNA signature for improving outcomes in colorectal cancer
Source: Nat Commun. 2022 Feb 10;13:816. doi: 10.1038/s41467-022-28421-6 (PMC8831564; doi:10.1038/s41467-022-28421-6)
Supplement: Supplementary file 9 — Reporting Summary [file 41467_2022_28421_MOESM9_ESM.pdf]

nature portfolio

Corresponding author(s): Xinwei Han, Zhenqiang Sun

Last updated by author(s): Dec 3, 2021

Reporting Summary

Nature Portfolio wishes to improve the reproducibility of the work that we publish. This form provides structure for consistency and transparency in reporting. For further information on Nature Portfolio policies, see our Editorial Policies and the Editorial Policy Checklist.

Statistics

For all statistical analyses, confirm that the following items are present in the figure legend, table legend, main text, or Methods section.

n/a	Confirmed	
☐	☒	The exact sample size (*n*) for each experimental group/condition, given as a discrete number and unit of measurement
☐	☒	A statement on whether measurements were taken from distinct samples or whether the same sample was measured repeatedly
☐	☒	The statistical test(s) used AND whether they are one- or two-sided<br>*Only common tests should be described solely by name; describe more complex techniques in the Methods section.*
☐	☒	A description of all covariates tested
☐	☒	A description of any assumptions or corrections, such as tests of normality and adjustment for multiple comparisons
☐	☒	A full description of the statistical parameters including central tendency (e.g. means) or other basic estimates (e.g. regression coefficient) AND variation (e.g. standard deviation) or associated estimates of uncertainty (e.g. confidence intervals)
☐	☒	For null hypothesis testing, the test statistic (e.g. *F*, *t*, *r*) with confidence intervals, effect sizes, degrees of freedom and *P* value noted<br>*Give P values as exact values whenever suitable.*
☒	☐	For Bayesian analysis, information on the choice of priors and Markov chain Monte Carlo settings
☐	☒	For hierarchical and complex designs, identification of the appropriate level for tests and full reporting of outcomes
☐	☒	Estimates of effect sizes (e.g. Cohen's *d*, Pearson's *r*), indicating how they were calculated

Our web collection on statistics for biologists contains articles on many of the points above.

Software and code

Policy information about availability of computer code

Data collection	No code was used to collect data in this study - code was only used for data analysis.
Data analysis	All data processing, statistical analysis, and plotting were conducted in R 4.0.5 software. R codes are deposited in GitHub: https://github.com/Zaoqu-Liu/IRLS.<br>survminer v0.4.9 package (https://cran.r-project.org/web/packages/survminer/index.html);<br>Affy v1.72.0 package (https://www.bioconductor.org/packages/release/bioc/html/affy.html);<br>GSVA v1.42.0 package (https://www.bioconductor.org/packages/release/bioc/html/GSVA.html);<br>ConsensusClusterPlus v1.58.0 package (https://www.bioconductor.org/packages/release/bioc/html/ConsensusClusterPlus.html);<br>WGCNA v1.70-3 package (https://cran.r-project.org/web/packages/WGCNA/index.html);<br>survival v3.2-13 package (https://cran.r-project.org/web/packages/survival/index.html);<br>CompareC v1.3.1 package (https://cran.r-project.org/web/packages/compareC/index.html);<br>pROC v1.18.0 package (https://cran.r-project.org/web/packages/pROC/index.html);<br>timeROC v0.4 package (https://cran.r-project.org/web/packages/timeROC/index.html);<br>risksetROC v1.0.4 package (https://cran.r-project.org/web/packages/risksetROC/index.html);<br>CMSclassifier v1.0.0 package (https://github.com/Sage-Bionetworks/CMSclassifier)

For manuscripts utilizing custom algorithms or software that are central to the research but not yet described in published literature, software must be made available to editors and reviewers. We strongly encourage code deposition in a community repository (e.g. GitHub). See the Nature Portfolio guidelines for submitting code & software for further information.

Data

Policy information about availability of data

All manuscripts must include a data availability statement. This statement should provide the following information, where applicable:

- Accession codes, unique identifiers, or web links for publicly available datasets
- A description of any restrictions on data availability
- For clinical datasets or third party data, please ensure that the statement adheres to our policy

Public data used in this work can be acquired from the TCGA Research Network portal (https://portal.gdc.cancer.gov/) and Gene Expression Omnibus (GEO, http://www.ncbi.nlm.nih.gov/geo/).
TCGA-CRC: https://portal.gdc.cancer.gov/
GSE17536: https://www.ncbi.nlm.nih.gov/geo/query/acc.cgi?acc=GSE17536
GSE17537: https://www.ncbi.nlm.nih.gov/geo/query/acc.cgi?acc=GSE17537
GSE29621: https://www.ncbi.nlm.nih.gov/geo/query/acc.cgi?acc=GSE29621
GSE38832: https://www.ncbi.nlm.nih.gov/geo/query/acc.cgi?acc=GSE38832
GSE39582: https://www.ncbi.nlm.nih.gov/geo/query/acc.cgi?acc=GSE39582
GSE72970: https://www.ncbi.nlm.nih.gov/geo/query/acc.cgi?acc=GSE72970
GSE31595: https://www.ncbi.nlm.nih.gov/geo/query/acc.cgi?acc=GSE31595
GSE92921: https://www.ncbi.nlm.nih.gov/geo/query/acc.cgi?acc=GSE92921
GSE143985: https://www.ncbi.nlm.nih.gov/geo/query/acc.cgi?acc=GSE143985
GSE161158: https://www.ncbi.nlm.nih.gov/geo/query/acc.cgi?acc=GSE161158
GSE19860: https://www.ncbi.nlm.nih.gov/geo/query/acc.cgi?acc=GSE19860]
GSE19862: https://www.ncbi.nlm.nih.gov/geo/query/acc.cgi?acc=GSE19862
GSE28702: https://www.ncbi.nlm.nih.gov/geo/query/acc.cgi?acc=GSE28702
GSE62080: https://www.ncbi.nlm.nih.gov/geo/query/acc.cgi?acc=GSE62080
GSE69657: https://www.ncbi.nlm.nih.gov/geo/query/acc.cgi?acc=GSE69657
GSE45404: https://www.ncbi.nlm.nih.gov/geo/query/acc.cgi?acc=GSE45404
GENCODE: https://www.gencodegenes.org/

Field-specific reporting

Please select the one below that is the best fit for your research. If you are not sure, read the appropriate sections before making your selection.

[x] Life sciences [ ] Behavioural & social sciences [ ] Ecological, evolutionary & environmental sciences

For a reference copy of the document with all sections, see nature.com/documents/nr-reporting-summary-flat.pdf

Life sciences study design

All studies must disclose on these points even when the disclosure is negative.

Sample size	The datasets involved in this study was determined by specific clincial information (e.g., overall survival, relapse-free survival, fluorouracil treatment and bevacizumab treatment) and the same annotation platform (the Affymetrix® GPL570 platform). The sample sizes were determined based on previous publications.
Data exclusions	no data was excluded.
Replication	All attempts at replication were successful. How many times each experiment was performed and which statistical analysis used is indicated in the figure legends.
Randomization	Samples were allocated to groups based on the immune-related lncRNA signature score, immune status, and drug-sensitivity if applicable.
Blinding	This study aimed to systematically established a novel score system link to immune-derived lncRNAs to optimize precision treatment and further improve the clinical outcomes of individual patients. Thus blinding was not relevant.

Reporting for specific materials, systems and methods

We require information from authors about some types of materials, experimental systems and methods used in many studies. Here, indicate whether each material, system or method listed is relevant to your study. If you are not sure if a list item applies to your research, read the appropriate section before selecting a response.

Materials & experimental systems

n/a	Involved in the study
☐	☒ Antibodies
☒	☐ Eukaryotic cell lines
☒	☐ Palaeontology and archaeology
☒	☐ Animals and other organisms
☐	☒ Human research participants
☒	☐ Clinical data
☒	☐ Dual use research of concern

Methods

n/a	Involved in the study
☒	☐ ChIP-seq
☒	☐ Flow cytometry
☒	☐ MRI-based neuroimaging

Antibodies

Antibodies used

Antibodies are listed below as: target label (dilution; catalog number; company):
CD8A (1:300; Cat# GB13068-2; Servicebio, Wuhan, China)
PD-L1 (1:500; Cat# GB11339; Servicebio, Wuhan, China)

Validation

Each antibody specificity was validated by the manufacture, and the validation result is posted on their website.

Human research participants

Policy information about studies involving human research participants

Population characteristics

A total of 232 frozen surgically resected CRC tissues were collected from The First Affiliated Hospital of Zhengzhou University. Of which, 101 patients ≤65 years old and 131 patients >65 years old, 126 males and 106 females, 40 patients in T1/2 and 192 patients in T3/4, 177 patients in N0 and 55 patients in N1/2, 203 patients in M0 and 29 patients in M1, 122 patients in AJCC stage I/II and 110 patients in AJCC stage III/IV, 139 patients in MSI-L/MSS and 65 patients in MSI-H, 62 patients in dead status, 80 patients with relapse, 88 patients treated with adjutant chemotherapy, 53 nonresponders and 35 responders to adjutant chemotherapy, 65 patients treated with pembrolizumab, and 42 nonresponders and 23 responders to pembrolizumab.

Recruitment

All patients gave written informed consent, received available standard systemic therapies (fluorouracil, oxaliplatin, irinotecan, and pembrolizumab); were aged 18 years or older; had adequate hematologic, renal, and liver function; had Eastern Cooperative Oncology Group (ECOG) performance status of 0 or 1, and measurable disease according to Response Evaluation Criteria in Solid Tumors (RECIST, version 1.1). Responders and nonresponders were defined as complete response (CR)/partial response (PR) and stable disease (SD)/progressive disease (PD), respectively.

Ethics oversight

The human cancer tissues used in this study were approved by Ethnics Committee of The First Affiliated Hospital of Zhengzhou University on December 19, 2019, and the TRN is 2019-KW-423.

Note that full information on the approval of the study protocol must also be provided in the manuscript.
